# Clinical Efficacy and Safety of Aidi Injection Plus Docetaxel-Based Chemotherapy in Advanced Nonsmall Cell Lung Cancer: A Meta-Analysis of 36 Randomized Controlled Trials

**DOI:** 10.1155/2018/7918258

**Published:** 2018-06-11

**Authors:** Zheng Xiao, Chengqiong Wang, Lianhong Li, Xuemei Tang, Nana Li, Jing Li, Ling Chen, Qihai Gong, Fushan Tang, Jihong Feng, Xiaofei Li

**Affiliations:** ^1^Evidence-Based Medicine Center, MOE Virtual Research Center of Evidence-Based Medicine at Zunyi Medical College, Affiliated Hospital of Zunyi Medical College, Zunyi 563003, Guizhou, China; ^2^Department of Respiratory Medicine (Center for Evidence-Based and Translational Medicine of Major Infectious Diseases), Affiliated Hospital of Zunyi Medical College, Zunyi 563003, Guizhou, China; ^3^Project Team of Health Management and Decision-Making, Health Education Society in Zunyi, Zunyi 563002, Guizhou, China; ^4^Department of Neurology, First People's Hospital of Zunyi City and Third Affiliated Hospital of Zunyi Medical College, Zunyi 563002, Guizhou, China; ^5^School of pharmacy, Zunyi Medical College, Zunyi 563003, Guizhou, China; ^6^Department of Oncology, Affiliated Hospital of Zunyi Medical College, Zunyi 563000, Guizhou, China; ^7^Special Key Laboratory of Special Antitumor Drugs of Guizhou Province, Zunyi Medical College, Zunyi 563003, Guizhou, China

## Abstract

*Background. *Aidi injection is an important adjuvant anticancer drug commonly used in China. Can Aidi injection plus docetaxel-based chemotherapy improve clinical efficacy with good safety in NSCLC? To further reveal its clinical effectiveness, we systematically evaluated all the related studies.* Method. *We collected all the studies about Aidi injection plus docetaxel-based chemotherapy for NSCLC on Medline, Embase, Web of Science, CNKI, VIP, Wanfang, CBM,* CENTRAL, *Chi-CTR, and US-clinical trials. We evaluated their methodological bias risk according to the Cochrane evaluation handbook (5.1.0), extracted data following the predesigned data extraction form according to the PICO principle, and synthesized the data* using* meta-analysis.* Results. *We included 36 RCTs with 2837 patients, and most studies had unclear bias risk. The merged RR values and their 95% CI of meta-analysis for ORR, DCR, and QOL were as follows: 1.30 (1.19, 1.42), 1.17, (1.12, 1.22), and 1.73 (1.54, 1.95). The merged RR values for neutropenia, thrombocytopenia, anemia, gastrointestinal toxicity, hepatorenal dysfunctions, and alopecia were as follows: 0.70 (0.61, 0.79), 0.63 (0.53, 0.75), 0.60 (0.48, 0.75), 0.76 (0.65, 0.89), 0.56 (0.36, 0.88), and 0.58 (0.36, 0.93).* Compared with chemotherapy alone, *all differences were statistically significant. Subgroup analysis showed that, with 100 ml, 80-100 ml, and 50 ml, Aidi injection could increase the tumor response and Aidi injection plus DP, DC, and DO could increase the tumor response. Meta-analysis results had good stability.* Conclusions. *Aidi injection plus docetaxel-based chemotherapy, especially plus DP, DC, and DO, may significantly improve the clinical efficacy and QOL in NSCLC.* It may also have low risk of hematotoxicity, gastrointestinal toxicity, and low risk of inducing hepatorenal dysfunctions. *Aidi injection may have attenuation and synergistic efficacy to docetaxel chemotherapy.* All these need to have new evidence to be proved*.

## 1. Introduction

Lung cancer is the leading cause of cancer-related mortality around the world with* only 15*%* of 5-years survival rate *[1–3]. Approximately 80% of lung cancers are nonsmall cell lung cancer (NSCLC). Nevertheless, over 50% of patients with NSCLC have advanced local invasion and metastasis, when they were admitted to* the* hospital for diagnosis. They must* receive* the systemic chemotherapy, radiotherapy, or chemoradiotherapy* because they missed the opportunity for operation* [4–6]. As first- or second-line chemotherapy,* taxane agents including paclitaxel (taxol) and docetaxel (taxotere)* are widely used in NSCLC. But they have different acute/subacute toxicity, which results in poor prognosis with only 15% of 5-years survival rate and substandard quality of life (QOL) [7, 8]. Therefore, new effective strategies with attenuation and synergistic efficacy are urgently needed.

As Cantharis and Astragalus-based Chinese herbs, Aidi injection (Z52020236, China Food and Drug Administration) is composed of the extracts of Cantharis, Astragalus, Eleutherococcus senticosus, and Ginseng, which appear to have antitumor efficacy and reduce the toxicity [9–13]. Meta-analysis (Wang, Q. 2010) [14] reported that Aidi injection plus paclitaxel or docetaxel and cisplatin could significantly improve the clinical efficiency and QOL in NSCLC. The combination had low risk of neutropenia, thrombocytopenia, and nausea/vomiting,* but unclear risk *of anemia, hepatotoxicity, nephrotoxicity, neurotoxicity, and alopecia. However, many studies [15–18] showed that docetaxel and paclitaxel had different clinical manifestations, especially the acute/subacute toxicity. Docetaxel is one of the important first- or second-line chemotherapeutic agents for NSCLC [19–21]. And docetaxel-based chemotherapy refers to docetaxel alone or plus cisplatin, carboplatin, oxaliplatin, lobaplatin,* or *nedaplatin, which are important chemotherapy regimens in NSCLC. The application of Aidi injection plus docetaxel-based chemotherapy was clinically* used* in a wide range of* treatment. *Can Aidi injection plus docetaxel-based chemotherapy improve clinical efficacy with* satisfying level of safety *in NSCLC? Has Aidi injection attenuated and synergistic efficacy to docetaxel-based chemotherapy in NSCLC? Many studies [22–25] had shown that Aidi injection plus docetaxel*-*based chemotherapy might improve the clinical efficacy and QOL with low* risk of *acute/subacute toxicity in NSCLC. However, these conclusions* vary* in different studies with limited sample size. At present, there is a* lack *of strong evidence to* prove the efficacy of the treatments*. Therefore, to further reveal its real clinical* efficacy* and provide the best evidence for clinical strategies in NSCLC, we systematically evaluated all the related studies.

## 2. Materials and Methods

This article followed Preferred Reporting Items for Systematic Reviews and Meta-Analyses guidelines (PRISMA guidelines). Ethical approval was not required, as materials of this study* were *published or unpublished studies.

### 2.1. Search Strategy

Two reviewers (Chengqiong Wang and Lianhong Li) independently searched articles in Chinese and English databases using the search strategy (Aidi OR Aidi injection OR Compound cantharis injection OR Compound disodium cantharidinate injection or Addie injection) and the search strategy (Taxoids OR Docetaxel OR Docetaxel OR Taxotere) and the search strategy (“Lung Neoplasms”[Mesh] OR Lung cancer OR Lung cancers OR Non small cell lung cancer OR NSCLC OR SCLC OR Pulmonary neoplasms OR Lung neoplasm OR Pulmonary neoplasm OR Pulmonary* cancer* OR Pulmonary cancers OR Lung carcinoma OR Pulmonary carcinoma). Published studies were retrieved in Medline, Embase, Web of Science (ISI), China National Knowledge Infrastructure Database (CNKI), Chinese Scientific Journals Full-Text Database (VIP), Wanfang Database, China Biological Medicine Database (CBM) (established to September 2017), and Cochrane Central Register of Controlled Trials (CENTRAL, Issue 8 of 12, August 2017). Ongoing studies were retrieved in Chinese clinical trial registry (Chi-CTR) and US-clinical trials (established to September 2017). All retrievals were implemented by* using *the Mesh and free word. Finally, all related systematic reviews (SRs) or meta-analysis was evaluated, and studies meeting inclusion criteria were selected from the references.

### 2.2. Inclusion and Exclusion Criteria

Included studies must meet the following criteria. (1) The patients* had *NSCLC with stages III to IV being diagnosed and confirmed with the histopathological and cytological diagnostic criteria and TNM staging system. (2) There was no severe damage in liver or kidney function in any of the patients. (3) There were randomized controlled trials (RCTs). (4) The experimental group* undergone* Aidi injection plus docetaxel-based chemotherapy, and* the* control group* undergone* docetaxel-based chemotherapy. Docetaxel-based chemotherapy refers to docetaxel alone or plus platinum such as cisplatin, carboplatin, oxaliplatin, lobaplatin, and nedaplatin (DP, DC, DO, DL, and DN). (5) Patients* prior to being included *in the study* have not accepted the* radiotherapy, other chemotherapy, or Chinese herbs. (6) Main outcomes included the clinical efficacy and acute/subacute toxicity. Clinical efficacy was evaluated using tumor responses and QOL. (7) No restrictions were set on the follow-up time or types of hospitals.

Excluded studies must meet the following criteria: (1) duplicates, (2) unrelated studies including studies* concerning *Aidi injection plus paclitaxel chemotherapy, radiotherapy,* additional *chemotherapeutic agents, other Chinese herbs and other themes, (3) non-RCTs including case control studies and series case reports, (4) abstracts* and *reviews without specific data and unrelated SRs, and (5) studies without the clinical efficacy, QOL,* and* acute/subacute toxicity.

### 2.3. Bias Risk Assessment

According to the Cochrane evaluation handbook of RCTs (5.1.0) [26], we evaluated the bias risk of all trials using the bias parameters such as the random sequence generation (selection bias), the allocation concealment (selection bias), the blinding of participants and personnel (performance bias), the blinding of outcome assessment (detection bias), the incomplete outcome data (attrition bias), the selective reporting (reporting bias), and the other bias (whether the baseline is comparable). We judged each parameter on three levels (“yes” for a low risk of bias, “no” for a high risk of bias, and “unclear”). Then, we assessed the trials and categorized them into three levels: low risk (all items were “yes”), high risk (at least one item was “no”), and unclear risk (at least one item was “unclear”).

### 2.4. Selection and Evaluation of Studies

Two reviewers (Xuemei Tang and Nana Li) independently screened and assessed studies according to the above standards. Any disagreements were eliminated by discussing between themselves or with Zheng Xiao.

### 2.5. Main Outcomes

We measured the tumor response using objective response rate (ORR) and disease control rate (DCR). According to the World Health Organization (WHO) guidelines for solid tumor responses [27] or Response Evaluation Criteria in Solid Tumors (RECIST) [28], indicators were complete response (CR), partial response (PR), no change (NC), progressive disease (PD), ORR being equal to CR plus PR, and DCR being equal to CR plus PR and NC. According to Karnofsky Performance Status scale (KPS scale) [29, 30], QOL was considered to be improved if KPS score increased 10 points or higher after treatment. We measured the acute/subacute toxicity using hematotoxicity such as neutropenia (granulocytes < 2 × 10^9^/L), thrombocytopenia (platelets < 100 × 10^9^/L) and anemia (Hemoglobin < 110g/L), liver dysfunction (serum aminotransferase or alkaline phosphatase > 1.25 × N), renal dysfunction (serum urea nitrogen or creatinine > 1.25 × N), hepatorenal dysfunctions, and gastrointestinal toxicity including the gastrointestinal reactions and nausea/vomiting, neurotoxicity (peripheral neuritis), alopecia, rash, phlebitis, and oral mucositis.

### 2.6. Data Extraction

Two reviewers (Chengqiong Wang and Lianhong Li) independently extracted all the data in a predesigned data extraction form according to the PICO principle. All the data included the first author, the publishing time, the randomization methods, the demographic characteristics, the sample size, the usage of Aidi injection and the types of docetaxel chemotherapy, the evaluation criteria of clinical efficacy and acute or subacute toxicity and the follow-up* information*, and main outcomes including the ORR, DCR, QOL, and acute or subacute toxicity. The data were obtained directly from the articles. If insufficient details were reported, authors were contacted for further information.

### 2.7. Statistical Analysis

Meta-analysis was implemented by two reviewers (Chengqiong Wang and Jing Li) using Review Manager 5.3 (The Cochrane Collaboration, Oxford, UK). The relative risk (RR) and 95% confidence intervals (CI) were calculated. Statistical heterogeneity of the results across trials was assessed by chi-square based Q-statistic test and the consistency was calculated by I^2^. If the homogeneity (P ≥ 0.1, I^2^ ≤ 50%) was not rejected, the fixed-effects model (FEM) was used to calculate the summary RR and the 95% CI. Otherwise, the results were* calculated* by random-effects model (REM). We performed the subgroup analysis according to different doses of Aidi injection, docetaxel-based chemotherapy and evaluation criteria, which revealed their influence on the tumor responses. Publication bias was evaluated* using* funnel plots* if there were more than 10 included studies. *The poor* quality *studies and studies with over- or underestimated results were important factors* that damage *the robustness of meta-analysis results.* The studies were defined as poor quality studies when they had at least one domain considered as high risk of bias. *The over- or underestimated studies were identified according to the result of funnel plots and heterogeneity analysis, in which* results were statistically different and had positive effects on* publication bias or heterogeneity. Therefore, the sensitivity was evaluated through excluding the* poor quality studies* and studies with overestimated efficacy and underestimated toxicity.

## 3. Results

### 3.1. Search Results

**Figure 1:**
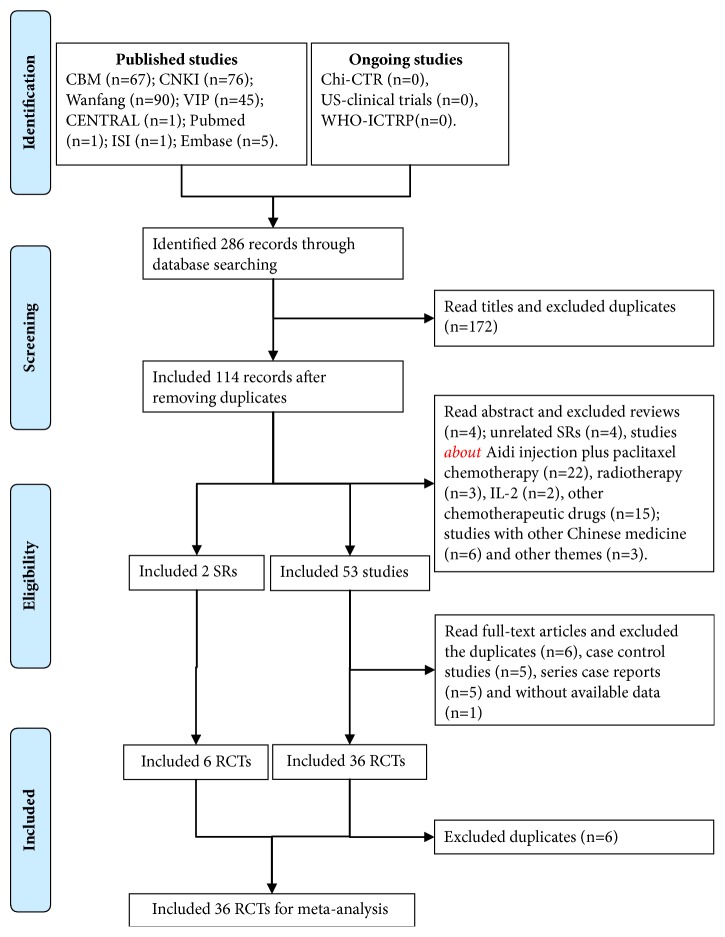
Articles retrieved and assessed for eligibility.

The initial database search identified 286 published studies without ongoing studies using our search strategies (Figure 1). Reading the title and excluding the duplicates, 114 records were included. After reading the abstract,* 51 full texts *and 2 SRs [14, 31] were included. And then reading the full text and 17 unqualified studies* excluded*, 36 RCTs [22–24, 32–64] were included.* After* further evaluating the 2 SRs [14, 31], 6 RCTs [22, 32–34, 36, 37] were included. Finally, we included 36 RCTs [22–24, 32–64] after excluding 6 RCTs from SRs.

### 3.2. Characteristics of Included Studies

In this meta-analysis, we included 36 RCTs [22–24, 32–64] with 2837 advanced NSCLC patients (Table 1). Docetaxel-based chemotherapy included docetaxel alone, DP, DC, DO, DL, and DN. Experimental group was Aidi injection plus docetaxel-based chemotherapy involving 1422 cases, and control group was docetaxel chemotherapy alone involving 1415 cases. The males and females were 1722 and 1044 cases, respectively, with age between 27 and 82 years. The dosage of Aidi injection was 40 100 ml/day, and treatment time was 1-6 weeks/cycle with 1-6 cycles by intravenous injection. Outcomes were evaluated at 6-12 w after treatment. According to the WHO guidelines [27] for solid tumor responses or RECIST [65], tumor responses were evaluated in 34 studies [22–24, 32–55, 57–61, 63, 64] involving 2714 patients. QOL was evaluated in 22 studies [22–24, 32–42, 44, 46–48, 56–59] involving 1676 patients. According to WHO standards [27] or National Cancer Institute Common Toxicity Criteria (NCI-CTC) [66], acute or subacute toxicity was evaluated in 31 studies [22, 23, 32–43, 45–47, 49–55, 57, 59–64] involving 2434 patients.

### 3.3. Methodological Bias Risk

**Figure 2:**
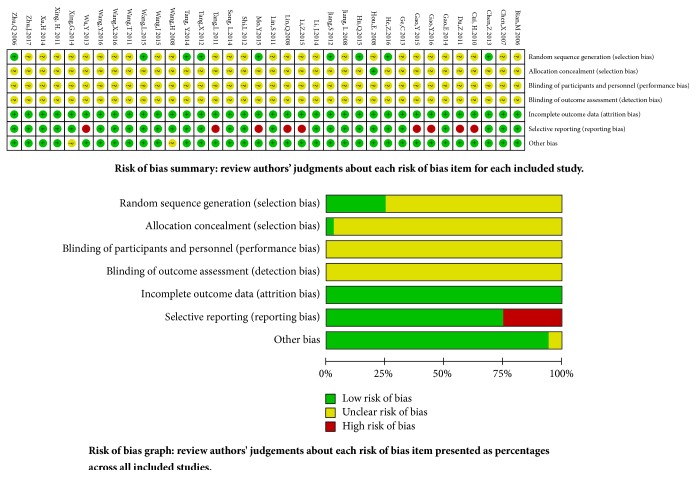
Risk of methodological bias.

In 36 studies,* nine studies *described the random sequence generation using randomized digital table* in eight studies* [33, 43, 45, 51, 55, 57, 59, 61] and* lottery in one study *[24]. The random allocation concealment was implemented using envelope in one study [34],* and other *studies did not provide the detailed information about it.* None of the studies *did provide the detailed information about blinding of participants, personnel, and outcome assessment. All studies had complete outcome data without loss to follow-up. Nine studies [23, 36, 38, 40, 47, 54, 56, 57, 60] had selective reporting about the acute/subacute toxicity. Except for two studies [37, 52], baseline* was* comparable in other studies.* The methodological bias risk* of all included studies is presented in Figure 2.

### 3.4. Tumor Response

**Figure 3:**
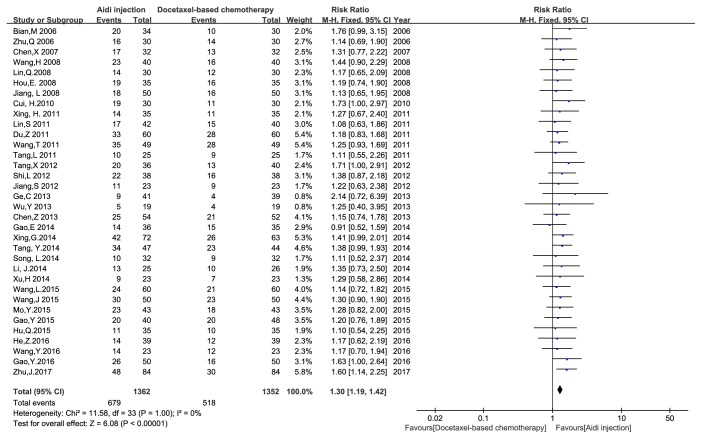
The analysis of ORR between two groups.

**Figure 4:**
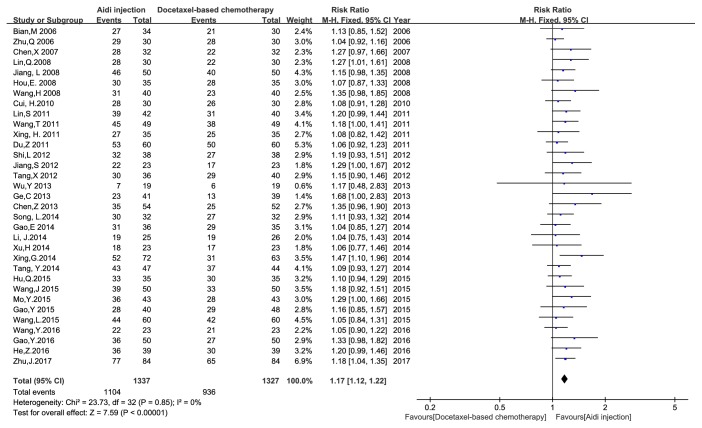
The analysis of DCR between two groups.

Thirty-four studies with 2714 cases [22–24, 32–55, 57–61, 63, 64] were reported the ORR (Figure 3). Pearson's chi-square test and I^2^ test showed that there was no statistical heterogeneity among studies (I^2^ = 0%). Meta-analysis showed that the ORR had* statistical differences* between Aidi injection plus docetaxel-based chemotherapy and docetaxel-based chemotherapy alone [RR = 1.30, 95% CI (1.19, 1.42), and P < 0.00001] by FEM. Thirty-three studies with 2664 cases reported the DCR (Figure 4). There was no statistical heterogeneity between studies (I^2^ = 0%). Meta-analysis showed that the DCR had* statistical differences* between the two groups [RR = 1.17, 95% CI (1.12, 1.22), and P < 0.00001] by FEM.

### 3.5. QOL

**Figure 5:**
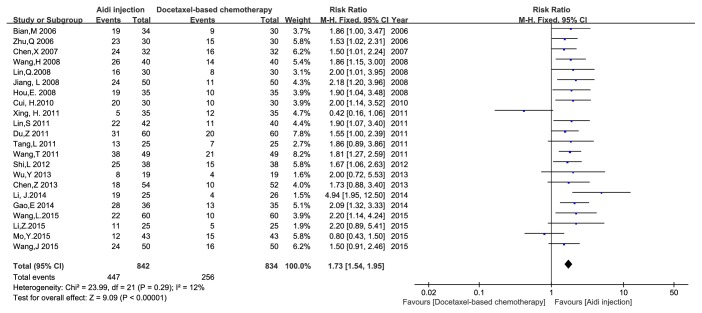
The analysis of QOL between two groups.

*The QOL was evaluated according to KPS scale *[29, 30]. Twenty-two studies with 1676 cases reported the QOL (Figure 5). There was minimal heterogeneity among studies (I^2^ = 12%). Meta-analysis showed that the QOL had* statistical differences *between the two groups [RR = 1.73, 95% CI (1.54, 1.95), and P < 0.00001] by FEM.

### 3.6. Acute/Subacute Toxicity

Thirty-one studies [22, 23, 32–43, 45–47, 49–55, 57, 59–64] involving 2434 patients reported the acute or subacute toxicity. There was heterogeneity among studies in neutropenia (I^2^ = 73%), gastrointestinal toxicity (I^2^ = 88%) and neurotoxicity (I^2^ = 56%), minimal heterogeneity in rash (I^2^ = 2%), and no heterogeneity in others toxicity (I^2^ = 0%). Meta-analysis showed that Aidi injection plus docetaxel-based chemotherapy had lower risk of neutropenia [RR = 0.70, 95% CI (0.61, 0.79), and P < 0.00001] and gastrointestinal toxicity [RR = 0.76, 95% CI (0.65, 0.89), and P = 0.0006] than* that of *docetaxel-based chemotherapy alone using REM and lower risk of thrombocytopenia [RR = 0.63, 95% CI (0.53, 0.75), and P < 0.00001], anemia [RR = 0.60, 95% CI (0.48, 0.75), and P < 0.00001], hepatorenal dysfunctions [RR = 0.56, 95% CI (0.36, 0.88), and P = 0.01], and alopecia [RR = 0.58, 95% CI (0.36, 0.93), and P = 0.02] than* that of *control group using FEM. And all differences were statistically significant (Table 2 and Figures S1, S2, S3, S4, S5, and S7). There were no statistical differences in liver dysfunction [RR = 0.69, 95% CI (0.47, 1.01), and P = 0.05], renal dysfunction [RR = 0.56, 95% CI (0.31, 1.00), and P = 0.05], neurotoxicity [RR = 0.65, 95% CI (0.35, 1.18), and P = 0.16], rash [RR = 0.75, 95% CI (0.38, 1.49), and P = 0.42], phlebitis [RR = 1.00, 95% CI (0.63, 1.59), and P = 1.00], and oral mucositis [RR = 0.64, 95% CI (0.38, 1.09), and P = 0.10] between the two groups (Table 2 and Figures S5, 6, and 7).

### 3.7. Subgroup Analysis of ORR and DCR

Subgroup analysis was performed to reveal the influence of different doses, docetaxel chemotherapy protocols, and evaluation criteria on the ORR and DCR. Drug doses included Aidi injection with 100 ml, 80-100 ml, 80 ml, 60 ml, 50 ml, and 40 ml/time. Subgroup analysis showed that, with 100 ml, 80-100 ml, and 50 ml, Aidi injection could increase the ORR and DCR (Table 3 and Figures S8-9). Docetaxel-based chemotherapy included docetaxel alone, DP, DC, DO, DL, and DN. Subgroup analysis showed that only Aidi injection plus DP, DC, and DO could increase the ORR and DCR (Table 3 and Figures S10-11). Tumor responses were evaluated using WHO or RECIST criteria. Subgroup analysis showed that Aidi injection plus docetaxel-based chemotherapy could increase the ORR and DCR using the WHO or RECIST criteria (Table 3 and* Figures* S8-13).

### 3.8. Publication Bias Analysis

**Figure 6:**
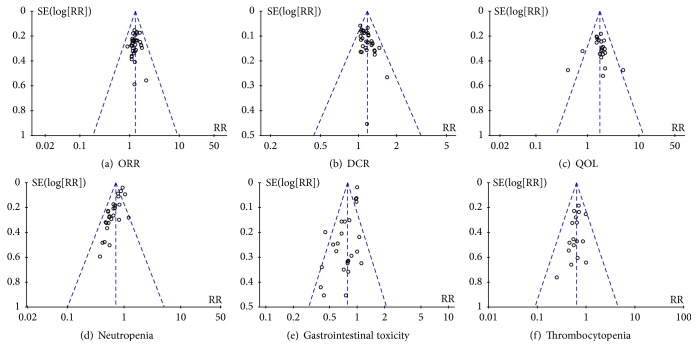
Publication bias analysis.

The funnel plots were symmetric in ORR and thrombocytopenia (Figures 6(a) and 6(f)). And there was no publication bias in these studies which objectively reported the results. The funnel plots were asymmetric in DCR, QOL, neutropenia, and gastrointestinal toxicity (Figures 6(b), 6(c), 6(d), and 6(e)). These results indicated that there was publication bias in them. The DCR was underestimated in one study [33]. The QOL was overestimated in one study [49] and underestimated in two studies [52, 57]. The neutropenia was overestimated in four studies [33, 35, 54, 59] and the gastrointestinal toxicity was overestimated in four studies [35, 39, 52, 59] and underestimated in one study [41].

### 3.9. Sensitivity Analysis

Nine* poor quality *studies [23, 36, 38, 40, 47, 54, 56, 57, 60] had at least one domain considered as high risk of bias and selective reporting about acute/subacute toxicity (Table 4(a)). They had potential effect on robustness of neutropenia, thrombocytopenia, gastrointestinal toxicity, and oral mucositis. Therefore, the sensitivity was evaluated through excluding poor* quality* studies. After excluding poor* quality* studies, all results had good consistency. There was statistical heterogeneity in neutropenia, gastrointestinal toxicity and neurotoxicity, and minimal heterogeneity in QOL. There was publication bias in DCR, QOL, neutropenia, and thrombocytopenia. Therefore, the sensitivity was evaluated through excluding the studies with overestimated efficacy or underestimated toxicity. Before and after excluding these studies, results had good consistency (Table 4(b)). In all, this meta-analysis had good stability.

## 4. Discussion

Based on previous meta-analysis [14, 31], we* eventually* included 36 RCTs involving 2837 patients with advanced NSCLC.* There were 1722 males and 1044 females, respectively,* with ages between* 27 and 82 years.* The usage of Aidi injection was 50 ml-100 ml/day, 2-3 weeks/cycle with 2-3 cycles by intravenous injection. Docetaxel-based chemotherapy included docetaxel alone, DP, DC, DO, DL, and DN. The tumor responses, QOL, and acute or subacute toxicity were evaluated at 6-12 w after treatment.

Docetaxel-based chemotherapy is important first- or second-line chemotherapeutic agents for NSCLC. Can Aidi injection plus docetaxel-based chemotherapy improve the clinical efficacy in NSCLC? Thirty-four studies [22–24, 32–55, 57–61, 63, 64] involving 2714 patients were included to evaluate the tumor responses. Meta-analysis showed that Aidi injection plus docetaxel-based chemotherapy could significantly improve the ORR and DCR in NSCLC. But there was significant clinical heterogeneity in them. Further subgroup analysis showed that Aidi injection with 100 ml, 80-100 ml, and 50 ml could increase the ORR and DCR and 50 ml was the main dosage. Combined with DP, DC, and DO, Aidi injection could increase the tumor responses. This meta-analysis involved 34 studies with 2714 cases which ensured sufficient sample size for analysis. The DCR was underestimated and the meta-analysis results had good robustness. All these* were* beneficial to* tumor responses*. But most studies had unclear* bias risk,* which weakened the result's reliability. Compared to the previous studies [14, 31], this meta-analysis revealed that Aidi injection plus docetaxel-based chemotherapy, especially plus DP, DC, and DO, might significantly improve the ORR and DCR and 50 ml was the main dosage. Our previous meta-analysis [67, 68] had shown that Aidi injection plus radiotherapy or gemcitabine and cisplatin (GP) could significantly improve the QOL in patients with lung cancer. Can Aidi injection plus docetaxel-based chemotherapy improve the QOL? To further analyze whether Aidi injection can improve the QOL, 22 studies with 1676 cases were included for analysis. Meta-analysis showed that Aidi injection could significantly improve the QOL. But, QOL was overestimated in one study [49] and underestimated in two studies [52, 57]. Sensitivity analysis revealed that QOL had good robustness. But most studies had unclear* bias risk.* Therefore, we believed that Aidi injection might also improve the QOL. Aidi injection is composed of extracts from Astragalus, Eleutherococcus senticosus, Ginseng, and Cantharis. In vitro studies [69–72] had shown that cantharidin could induce the tumor* cells'* apoptosis and inhibit the proliferation, migration, and invasion. Animal studies [73–75] had shown that cantharidin or Ginseng could significantly inhibit the growth of malignant tumor cells. Our previous meta-analysis [76] had revealed that Aidi injection could significantly restore the cellular immunity damaged by platinum-based chemotherapy. In addition, many studies [77, 78] had shown that Astragalus, senticosus Eleutherococcus, and Ginseng also had antitumor activity and immune regulation functions. These results provided indirect evidence for the above conclusions. In all, we believe that Aidi injection plus docetaxel-based chemotherapy, especially plus DP, DC, and DO, may significantly increase clinical efficacy and improve QOL in patients with NSCLC. The main dose may be 50 ml/time. Results indirectly indicate that Aidi injection may have synergistic efficacy to docetaxel-based chemotherapy. Unfortunately, So far, there was no reliable evidence to confirm the* long-term synergistic efficacy.*

Docetaxel-based chemotherapy has varying degrees of blood, liver, kidney, and gastrointestinal toxicity due to docetaxel plus platinum [79–81]. However, can Aidi injection plus docetaxel-based chemotherapy increase the risk of acute/subacute toxicity?* To answer this question,* 31 studies [22, 23, 32–43, 45–47, 49–55, 57, 59–64] involving 2434 patients were included to reveal the risk of toxicity. Meta-analysis showed that Aidi injection plus docetaxel-based chemotherapy had lower risk of the neutropenia, thrombocytopenia, anemia and gastrointestinal toxicity, hepatorenal dysfunctions, and alopecia* compared to that of *docetaxel-based chemotherapy alone. And there were no significant differences in liver dysfunction, renal dysfunction, neurotoxicity, rash, phlebitis, and oral mucositis between the two groups. The meta-analysis of neutropenia, thrombocytopenia, and gastrointestinal toxicity had sufficient studies and sample size. But there were limited studies and sample size in other meta-analysis, especially* in the meta-analysis of* liver and renal dysfunction, which might lead to insufficient assessment. Sensitivity analysis showed that the merged value of neutropenia, thrombocytopenia, and gastrointestinal toxicity had good robustness. Compared to the previous meta-analysis [14, 31], this study further revealed that Aidi injection plus docetaxel-based chemotherapy had* low risk of *the neutropenia, thrombocytopenia, and gastrointestinal toxicity. In addition,* we found that* it also had* low risk of *anemia, hepatorenal dysfunctions, and alopecia. Our previous study [67] had shown that Aidi injection plus GP had low risk of hematological and gastrointestinal toxicity and neurotoxicity in NSCLC. Furthermore, Aidi injection could alleviate the radiotherapy related toxicity, such as myelosuppression, radiation pneumonitis, and esophagitis [68]. These results provided indirect clinical evidence for the above conclusions. Zhu X and et al. [82, 83] had reported that Astragalus membranaceus injection (AMI) could promote myelopoiesis through improving the hematopoietic microenvironment and relieving the bone marrow cells apoptosis in mice. Hu, W et al. [84–87] had revealed that ginsenoside Rg1 also had antimyelotoxicity activity and promotion of myelopoiesis through enhancing the antioxidant and anti-inflammatory capacities of bone marrow mesenchymal stem cells (BMSCs) in vivo. Liu L and et.al [88] had shown that Astragalus injection ameliorated the cisplatin-induced nephrotoxicity through regulating the Bax and Bcl-2 expression in mice. Other study [89]* had shown* that ginsenoside Rg1 also had antioxidant activities which ameliorated the cisplatin-induced hepatic injury through Nrf2 signaling pathway in mice. All these revealed that Astragalus and Ginseng* could ameliorate chemotherapy related toxicity through enhancing* the antimyelotoxicity activity, antiapoptotic, and antioxidant activities. These results provided the basic and mechanism evidence for the above conclusions. In summary, Aidi injection plus docetaxel-based chemotherapy may have low risk of hematotoxicity, gastrointestinal toxicity, and hepatorenal dysfunctions. Based on the optimization of efficacy and safety, results indicated that the* optimal dose* might be 50 ml/time. These results indirectly reveal that Aidi injection may have attenuation* effect *to docetaxel related toxicity.

There were some limitations in this study. Firstly, Chinese and English databases were retrieved but not Japanese and Korean databases. All included studies were published in China, which* may lead* to ethnical bias. Secondly, only 9 studies reported the random allocation method.* No studies provided* the detailed information about the random allocation concealment and the binding. Nine studies had selective reporting about the acute/subacute toxicity. Third, long-term efficacy had not been evaluated. Fourth, most studies reported* the* acute/subacute toxicity using* WHO standards [27] or NCI-CTC [66]*. And there were limited studies and sample size in liver and renal dysfunction, neurotoxicity, and alopecia. All these limitations might lead to an inadequate assessment of the clinical efficacy and safety.

## 5. Conclusions

The available evidence indicates that Aidi injection plus docetaxel-based chemotherapy, especially plus DP, DC, and DO, may significantly improve the clinical efficacy and QOL in patients with NSCLC.* It may have low risk of hematotoxicity, gastrointestinal toxicity, and hepatorenal dysfunctions. *Results indirectly indicate that Aidi injection may have attenuation and synergistic efficacy to docetaxel chemotherapy. Based on the optimization of efficacy and safety, the results indicated that the* optimal dose* may be 50 ml/time. Unfortunately, whether Aidi injection can improve long-term efficacy is still unclear. Furthermore, many limitations might lead to an inadequate assessment* of* the clinical efficacy and safety. Therefore, we look forward to larger scale* RCTs* or real-world studies* for a more thorough review in* future publications.* Consequently, *we hope that this study will provide valuable evidence for Aidi injection as an important supplementary therapy for malignant tumors.

## Figures and Tables

**Table 1:** Characteristics of included studies.

**First author. year**	** NSCLC(III-IV)**			**Randomized Method**	**Interventions**		**C**	**Scale(A) **	**Scale(B) **	**Follow-up**	**Outcomes**
	**E/C**	**M/F**	**Age**		**E**	**Aidi(D/T/C)**					
Bian, M. 2006 [32]	34/30	44/20	30-75	Unclear	Aidi + DP	50 ml/15d/2	DP	WHO	WHO	8 w	O1, O2, O3
Zhu, Q. 2006 [33]	30/30	32/28	33-74	Randomized digital table	Aidi + DP	50 ml/10d/3	DP	RECIST	WHO	9 w	O1, O2, O3
Chen, X. 2007 [22]	32/32	39/25	47-72	Unclear	Aidi + DP	50 ml/15-20d/2	DP	WHO	WHO	6 w	O1, O2, O3
Hou, E. 2008 [34]	35/35	41/29	34-70	Unclear	Aidi + DP	50 ml/10d/2	DP	WHO	WHO	6 w	O1, O2, O3
Jiang, L. 2008 [35]	50/50	69/31	39-76	Unclear	Aidi + DP	50 ml/21d/-	DP	WHO	WHO	4 w	O1, O2, O3
Lin, Q. 2008 [36]	30/30	41/19	35-73	Unclear	Aidi + DP	50 ml/14d/2	DP	WHO	WHO	6 w	O1, O2, O3
Wang, H. 2008 [37]	40/40	51/29	30-70	Unclear	Aidi + DP	80-100 ml/14d/2	DP	WHO	WHO	6 w	O1, O2, O3
Cui, H. 2010 [38]	30/30	39/21	38-76	Unclear	Aidi + DP	80-100 ml/8w/1	DP	WHO	WHO	8 w	O1, O2, O3
Du, Z. 2011 [23]	60/60	94/26	42-71	Unclear	Aidi + DP	40 ml/20d/2	DP	WHO	WHO	8 w	O1, O2, O3
Lin, S. 2011 [39]	42/40	52/30	32-79	Unclear	Aidi + DP	50 ml/14d/2	DP	WHO	WHO	4 w	O1, O2, O3
Tang, L. 2011 [40]	25/25	28/22	37-74	Unclear	Aidi + DP	50 ml/14d/2	DP	RECIST	WHO	4 w	O1, O2, O3
Wang, T. 2011 [41]	49/49	65/33	30-78	Unclear	Aidi + DP	80-100 ml/14d/2	DP	WHO	WHO	6 w	O1, O2, O3
Xing, H. 2011 [42]	35/35	42/28	60-82	Unclear	Aidi + TXT	50 ml/15d/2	TXT	WHO	WHO	10 w	O1, O2, O3
Jiang, S. 2012 [43]	23/23	38/8	43-70	Randomized digital table	Aidi + DP	50 ml/14d/2	DP	WHO	WHO	6 w	O1, O3
Shi, L. 2012 [44]	38/38	55/21	38-72	Unclear	Aidi + DP	100 ml/14d/2	DP	WHO	No	6 w	O1, O2
Tang, X. 2012 [45]	36/40	42/34	38-73	Randomized digital table	Aidi + DP	50 ml/14d/2	DP	RECIST	WHO	6 w	O1, O3
Chen, Z. 2013 [24]	52/54	72/34	57-78	Lottery	Aidi + DP	50 mL/12W/-	DP	RECIST	No	12 w	O1, O2
Ge, C. 2013 [46]	41/39	52/28	53-77	Unclear	Aidi + DP	50 mL/4W/-	DP	WHO	WHO	4 w	O1, O3
Wu. Y. 2013 [47]	19/19	21/17	31-68	Unclear	Aidi + DP	60 ml/10d/2	DP	WHO	WHO	6 w	O1, O2, O3
Gao, E. 2014 [48]	36/35	37/34	Unclear	Unclear	Aidi + DP	50 ml/14d/3	DP	Unclear	No	9 w	O1, O2
Li, J. 2014 [49]	25/26	19/17	42-75	Unclear	Aidi + DO	100 ml/10d/3	DO	WHO	WHO	12 w	O1, O3
Song, L. 2014 [50]	32/32	41/23	27-75	Unclear	Aidi + DC	50 ml/21d/2	DC	WHO	NCI-CTC 2.0	6 w	O1, O3
Tang, Y. 2014 [51]	47/44	45/46	46-71	Randomized digital table	Aidi + DO	50 ml/14d/3	DO	WHO	NCI-CTC 3.0	3 y	O1, O3
Xing, G. 2014 [52]	72/63	92/43	Unclear	Unclear	Aidi + DL	100 ml/14d/1-6	DL	WHO	WHO	3 y	O1, O3
Xu, H. 2014 [53]	23/23	24/22	52-74	Unclear	Aidi + DP	50 ml/42d/2	DP	WHO	WHO	6 w	O1, O3
Gao, Y. 2015 [54]	40/48	59/29	32-78	Unclear	Aidi + DP	80 mL/14d/3	DP	RECIST	WHO	9 w	O1, O3
Li, Z. 2015 [56]	25/25	37/13	65-80	Unclear	Aidi + TXT	50 ml/10d/1	TXT	No	Unclear	Unclear	O2
Hu, Q. 2015 [55]	35/35	41/29	34-76	Randomized digital table	Aidi + DC	50 ml/14d/2	DC	WHO	WHO	6 w	O1, O3
Wang, J. 2015 [58]	50/50	58/42	35-76	Unclear	Aidi + DP	80-100 ml/14d/2	DP	WHO	No	6 w	O1, O2
Mo, Y. 2015 [57]	43/43	49/37	Unclear	Randomized digital table	Aidi + DP	50 ml/14d/1	DP	WHO	WHO	6 w	O1, O2, O3
Wang, L. 2015 [59]	60/60	74/46	62-78	Randomized digital table	Aidi + TXT	50 ml/21d/1	TXT	WHO	WHO	Unclear	O1, O2, O3
Gao, Y. 2016 [60]	50/50	54/36	32-78	Unclear	Aidi + DN	80 mL/14d/3	DN	RECIST	WHO	9 w	O1, O3
He, Z. 2016 [61]	39/39	27/51	46-70	Randomized digital table	Aidi + DC	-/14d/-	DC	WHO	WHO	Unclear	O1, O3
Wang, Y. 2016 [63]	23/23	Unclear	40-70	Unclear	Aidi + DP	50 ml/10d/2	DP	RECIST	WHO	6 w	O1, O3
Wang, X. 2016 [62]	37/36	53/20	Unclear	Unclear	Aidi + DP	100 ml/7d/2	DP	no	WHO	6 w	O3
Zhu, J. 2017 [64]	84/84	95/73	31-75	Unclear	Aidi + DC	50 ml/14d/1	DC	WHO	WHO	6 w	O1, O3

**Note:** NSCLC: nonsmall cell lung cancer; E/C: experimental group (Aidi injection plus docetaxel-based chemotherapy) /control group (docetaxel-based chemotherapy); M/F: male/female; Aidi (D/T/C): dose/time/cycles; TXT: docetaxel; DP: docetaxel and cisplatin; DC: docetaxel and carboplatin; DO: docetaxel and oxaliplatin; DL: docetaxel and lobaplatin; DN: docetaxel and nedaplatin; scale. A: evaluation criteria of tumor response; scale. B: evaluation criteria of acute/chronic toxicity; RECIST: response evaluation criteria in solid tumors; NCI-CTC: National Cancer Institute Common Toxicity Criteria; O: outcomes; O1: ORR and DCR; O2: QOL; O3: acute /chronic toxicity.

**Table 2:** Meta-analysis results of acute/chronic toxicity (Figures S1-7).

**Outcomes**	**Studies**	**Experimental group (Evens/tatol)**	**Control groups (Evens/tatol)**	**SM**	**RR (95**%** CI)**	**I** ^**2**^	**P**
Neutropenia (Figure S1)	26	452/1007	627/999	REM	0.70 [0.61, 0.79]	73%	P < 0.00001
Thrombocytopenia (Figure S2)	17	153/715	235/700	FEM	0.63 [0.53, 0.75]	0%	P < 0.00001
Anemia (Figure S3)	9	85/353	135/343	FEM	0.60 [0.48, 0.75]	0%	P < 0.00001
Gastrointestinal toxicity (Figure S4)	26	504/1060	634/1053	REM	0.76 [0.65, 0.89]	88%	P = 0.0006
Liver dysfunction (Figure S5)	7	37/308	52/293	FEM	0.69 [0.47, 1.01]	0%	P = 0.05
Renal dysfunction (Figure S5)	5	15/181	26/173	FEM	0.56 [0.31, 1.00]	0%	P = 0.05
Hepatorenal dysfunctions (Figure S5)	5	23/147	40/146	FEM	0.56 [0.36, 0.88]	0%	P = 0.01
Neurotoxicity (Figure S6)	5	42/192	66/184	REM	0.65 [0.35, 1.18]	56%	P = 0.16
Alopecia (Figure S7)	3	16/98	27/92	FEM	0.58 [0.36, 0.93]	0%	P = 0.02
Rash (Figure S7)	2	12/88	15/83	FEM	0.75 [0.38, 1.49]	2%	P = 0.42
Phlebitis (Figure S7)	3	25/113	25/113	FEM	1.00 [0.63, 1.59]	0%	P = 1.00
Oral mucositis (Figure S7)	3	18/110	28/110	FEM	0.64 [0.38, 1.09]	0%	P = 0.10

**Note:** SM: statistical method; REM: random-effects model; FEM: fixed-effects model; RR: risk ratios.

**Table 3:** Subgroup analysis results of ORR and DCR(Figures S8-13).

**Subgroups**	**Objective response rate (ORR)**						**Disease control rate (DCR)**					
	***Studies***	**Cases**	**SM**	**RR(95**%** CI)**	**I** ^**2**^	**p**	***Studies***	**Cases**	**SM**	**RR(95**%** CI)**	**I** ^**2**^	**P**
**Total **	34	2714	FEM	1.30 [1.19, 1.42]	0%	P < 0.00001	33	2664	FEM	1.17 [1.12, 1.22]	3%	P < 0.00001

**Different drugs and doses (Figures ** S8-9 **)**												

Aidi injection (100 ml)	3	262	FEM	1.39 [1.08, 1.80]	0%	P = 0.01	3	262	FEM	1.27 [1.07, 1.50]	27%	P = 0.005
Aidi injection (80-100 ml)	4	338	FEM	1.37 [1.13, 1.67]	0%	P = 0.002	4	338	FEM	1.19 [1.06, 1.33]	0%	P = 0.002
Aidi injection (80 ml)	2	188	FEM	1.40 [1.00, 1.95]	0%	P = 0.05	2	188	FEM	1.25 [1.00, 1.55]	0%	P = 0.05
Aidi injection (60 ml)	1	38	No	1.25 [0.40, 3.95]	No	P = 0.70	1	38	FEM	1.17 [0.48, 2.83]	No	P = 0.73
Aidi injection (50 ml)	22	1690	FEM	1.27 [1.14, 1.42]	4%	P < 0.0001	21	1640	FEM	1.16 [1.10, 1.21]	0%	P < 0.00001
Aidi injection (40 ml)	1	120	No	1.18 [0.83, 1.68]	No	P = 0.36	1	120	No	1.06 [0.92, 1.23]	No	P = 0.43
Aidi injection (Unclear)	1	78	No	1.17 [0.62, 2.19]	No	P = 0.63	1	78	FEM	1.20 [0.99, 1.46]	No	P = 0.07

**Different chemotherapy regimens (Figures ** S10-11 **)**												

Aidi injection plus DP	24	1767	FEM	1.27 [1.15, 1.41]	0%	P < 0.00001	23	1717	FEM	1.17 [1.12, 1.24]	0%	P < 0.00001
Aidi injection plus DC	4	380	FEM	1.36 [1.05, 1.76]	0%	P = 0.02	4	380	FEM	1.16 [1.07, 1.26]	0%	P = 0.0004
Aidi injection plus DO	2	142	FEM	1.37 [1.02, 1.85]	0%	P = 0.04	2	142	FEM	1.07 [0.93, 1.24]	0%	P = 0.35
Aidi injection plus DL	1	135	No	1.41 [0.99, 2.01]	No	P = 0.05	1	135	No	1.47 [1.10, 1.96]	No	P = 0.009
Aidi injection plus DN	1	100	No	1.63 [1.00, 2.64]	No	P = 0.05	1	100	No	1.33 [0.98, 1.82]	No	P = 0.07
Aidi injection plus docetaxel	2	190	FEM	1.19 [0.82, 1.73]	0%	P = 0.37	2	190	FEM	1.06 [0.89, 1.26]	0%	P = 0.52

**Different evaluation criteria (Figures ** S12-13 **)**												

WHO Criteria	27	2188	FEM	1.30 [1.18, 1.43]	0%	P < 0.00001	27	2188	FEM	1.17 [1.12, 1.22]	0%	P < 0.00001
RECIST	7	526	FEM	1.30 [1.07, 1.57]	0%	P = 0.008	6	476	FEM	1.18 [1.06, 1.32]	41%	P = 0.003

**Note:** DP: docetaxel and cisplatin; DC: docetaxel and carboplatin; DO: docetaxel and oxaliplatin; DL: docetaxel and lobaplatin; DN: docetaxel and nedaplatin; SM: statistical method; RR: *risk ratio; *FEM: fixed-effects model.

**Table:** (a) Sensitivity analysis by excluding the poor trials.

**Indicators**	**Number**	**SM**	**RR(95**%** CI)**	**I** ^**2**^	**Excluded studies**	**Number**	**SM**	**RR(95**%** CI)**	**I** ^**2**^
Neutropenia	26	REM	0.70 [0.61, 0.79]	73%	Poor*∗*[36, 47, 54, 57]	22	REM	0.70 [0.61, 0.80]	75%
Thrombocytopenia	17	FEM	0.63 [0.53, 0.75]	0%	Poor*∗*[57]	16	FEM	0.65 [0.55, 0.76]	0%
Gastrointestinal toxicity	26	REM	0.76 [0.65, 0.89]	88%	Poor*∗* [36, 38, 54, 57]	22	REM	0.75 [0.63, 0.89]	90%
Oral mucositis	3	FEM	0.64 [0.38, 1.09]	0%	Poor*∗*[57]	3	FEM	0.64 [0.38, 1.09]	0%

**Table:** (b) Sensitivity analysis excluding the under- or over-estimated trials.

**Indicators**	**Number**	**SM**	**RR(95**%** CI)**	**I** ^**2**^	**Excluded studies**	**Number**	**SM**	**RR(95**%** CI)**	**I** ^**2**^
DCR	33	FEM	1.17 [1.12, 1.22]	0%	Over*∗*[36, 52, 64]	30	FEM	1.16 [1.11, 1.21]	0%
QOL	22	FEM	1.73 [1.54, 1.95]	12%	Over*∗*[22, 33–39, 41, 44, 48, 49, 59]	9	FEM	1.41 [1.14, 1.74]	38%
Neutropenia	26	REM	0.70 [0.61, 0.79]	73%	Under*∗*[22, 34, 36, 39, 43, 49, 52, 57, 62, 64], Over*∗*[33, 35]	14	FEM	0.72 [0.63, 0.81]	29%
Thrombocytopenia	17	FEM	0.63 [0.53, 0.75]	0%	Under*∗*[52, 59]	15	FEM	0.66 [0.54, 0.79]	0%
Gastrointestinal toxicity	26	REM	0.76 [0.65, 0.89]	88%	Under*∗*[23, 33, 41, 42, 53, 63, 64], Over*∗*[35]	18	FEM	0.86 [0.79, 0.94]	5%
Neurotoxicity	5	REM	0.65 [0.35, 1.18]	56%	Under*∗*[41, 42]	3	FEM	1.04 [0.65, 1.68]	0%

**Note:** DCR: disease control rate; QOL: quality of life; FEM: fixed-effects model; REM: random-effects model; RR: relative risk; SM: statistical method; CI: confidence interval; poor trials (Poor*∗*) had at least one domain considered as high risk of bias; over*∗* or under*∗*: over- or underestimated trials of which results had statistical difference and positive effects on publication bias and heterogeneity.

## Abbreviations

AMI: Astragalus membranaceus injection
BMSCs: Bone marrow mesenchymal stem cells
CBM: China Biological Medicine Database
CENTRAL: Cochrane Central Register of Controlled Trials
CR: Complete response
Chi-CTR: Chinese clinical trial registry
CI: Confidence interval
CNKI: China National Knowledge Infrastructure Database
DC: Docetaxel and carboplatin
DCR: Disease control rate
DL: Docetaxel and lobaplatin
DN: Docetaxel and nedaplatin
DO: Docetaxel and oxaliplatin
DP: Docetaxel plus cisplatin
FEM: Fixed effect model
GP: Gemcitabine and cisplatin
ISI: Web of Science
KPS scale: Karnofsky Performance Status scale
NSCLC: Nonsmall cell lung cancer
NCI-CTC: National Cancer Institute Common Toxicity Criteria
NC: No change
ORR: Objective response rate
PD: Progressive disease
PR: Partial response
PRISMA guidelines: Preferred Reporting Items for Systematic Reviews and Meta-Analyses guidelines
QOL: Quality of life
RR: Relative risk
RCTs: Randomized controlled trials
RECIST: Response Evaluation Criteria in Solid Tumors
REM: Random-effects model
SRs: Systematic reviews
SM: Statistical Method
Taxol: Paclitaxel
TP: Paclitaxel plus cisplatin
VIP: Chinese Scientific Journals Full-Text Database
WANFANG: Wanfang Database
WHO: World Health Organization.

## Supplementary Materials

Supplementary Materials Meta-analysis results of acute or chronic toxicity (Figures S1-7). Figure S1: the analysis of neutropenia between the two groups. Meta-analysis showed that Aidi injection plus* docetaxel-based* chemotherapy had low risk of neutropenia [RR = 0.70, 95% CI (0.61, 0.79), and P < 0.00001] using random-effects model. Figure S2: the analysis of thrombocytopenia between the two groups. Meta-analysis showed that Aidi injection plus* docetaxel-based* chemotherapy had low risk of thrombocytopenia [RR = 0.63, 95% CI (0.53, 0.75), and P < 0.00001] using fixed-effects model. Figure S3: the analysis of anemia between the two groups. Meta-analysis showed that Aidi injection plus* docetaxel-based* chemotherapy had low risk of anemia [RR = 0.60, 95% CI (0.48, 0.75), and P < 0.00001] using fixed-effects model. Figure S4: the analysis of gastrointestinal toxicity between the two groups. Meta-analysis showed that Aidi injection plus* docetaxel-based* chemotherapy had low risk of gastrointestinal toxicity [RR = 0.76, 95% CI (0.65, 0.89), and P = 0.0006] using random-effects model. Figure S5: the analysis of hepatorenal dysfunctions between the two groups. Meta-analysis showed that Aidi injection plus* docetaxel-based* chemotherapy had low risk of hepatorenal dysfunctions [RR = 0.56, 95% CI (0.36, 0.88), and P = 0.01] using fixed-effects model. But there were no statistically significant differences in liver dysfunction [RR = 0.69, 95% CI (0.47, 1.01), and P = 0.05], renal dysfunction [RR = 0.56, 95% CI (0.31, 1.00), and P = 0.05] between two groups. Figure S6: the analysis of neurotoxicity between the two groups. There were no statistically significant differences in neurotoxicity [RR = 0.65, 95% CI (0.35, 1.18), and P = 0.16] between two groups. Figure S7: the analysis of other toxicity between the two groups. Meta-analysis showed that Aidi injection plus* docetaxel-based* chemotherapy had low risk of alopecia [RR = 0.58, 95% CI (0.36, 0.93), and P = 0.02] using fixed-effects model. But there were no statistically significant differences in rash [RR = 0.75, 95% CI (0.38, 1.49), and P = 0.42], phlebitis [RR = 1.00, 95% CI (0.63, 1.59), and P = 1.00], and oral mucositis [RR = 0.64, 95% CI (0.38, 1.09), and P = 0.10] between two groups. Subgroup analysis results of ORR and DCR (Figures S8-13). Figure S8: subgroup analysis of ORR via drug doses. Subgroup analysis showed that with 100 ml, 80-100 ml, and 50 ml, Aidi injection could all increase the ORR. Figure S9: subgroup analysis of DCR via drug doses. Subgroup analysis showed that with 100 ml, 80-100 ml and 50 ml, Aidi injection could all increase the DCR. Figure S10: subgroup analysis of ORR via docetaxel-based chemotherapy. Subgroup analysis showed that only Aidi injection plus DP, DC, and DO could increase the DCR. Figure S11: subgroup analysis of DCR via docetaxel-based chemotherapy. Subgroup analysis showed that only Aidi injection plus DP, DC, and DO could increase the DCR. Figure S12: subgroup analysis of ORR via evaluation criteria. Subgroup analysis showed that Aidi injection plus* docetaxel-based* chemotherapy could increase the ORR using the WHO or RECIST criteria. Figure S13: subgroup analysis of DCR via evaluation criteria. Subgroup analysis showed that Aidi injection plus* docetaxel-based* chemotherapy could increase the DCR using the WHO or RECIST criteria.
