# Supplementary material for: Clinical Efficacy and Safety of Aidi Injection Plus Docetaxel-Based Chemotherapy in Advanced Nonsmall Cell Lung Cancer: A Meta-Analysis of 36 Randomized Controlled Trials
Source: Evid Based Complement Alternat Med. 2018 Jun 11;2018:7918258. doi: 10.1155/2018/7918258 (PMC6016159; doi:10.1155/2018/7918258)
Supplement: Supplementary Materials — Meta-analysis results of acute or chronic toxicity (Figures S1-7). Figure S1: the analysis of neutropenia between the two groups. Meta-analysis showed that Aidi injection plus docetaxel-based chemotherapy had low risk of neutropenia [RR = 0.70, 95% CI (0.61, 0.79), and P < 0.00001] using random-effects model. Figure S2: the analysis of thrombocytopenia between the two groups. Meta-analysis showed that Aidi injection plus docetaxel-based chemotherapy had low risk of thrombocytopenia [RR = 0.63, 95% CI (0.53, 0.75), and P < 0.00001] using fixed-effects model. Figure S3: the analysis of anemia between the two groups. Meta-analysis showed that Aidi injection plus docetaxel-based chemotherapy had low risk of anemia [RR = 0.60, 95% CI (0.48, 0.75), and P < 0.00001] using fixed-effects model. Figure S4: the analysis of gastrointestinal toxicity between the two groups. Meta-analysis showed that Aidi injection plus docetaxel-based chemotherapy had low risk of gastrointestinal toxicity [RR = 0.76, 95% CI (0.65, 0.89), and P = 0.0006] using random-effects model. Figure S5: the analysis of hepatorenal dysfunctions between the two groups. Meta-analysis showed that Aidi injection plus docetaxel-based chemotherapy had low risk of hepatorenal dysfunctions [RR = 0.56, 95% CI (0.36, 0.88), and P = 0.01] using fixed-effects model. But there were no statistically significant differences in liver dysfunction [RR = 0.69, 95% CI (0.47, 1.01), and P = 0.05], renal dysfunction [RR = 0.56, 95% CI (0.31, 1.00), and P = 0.05] between two groups. Figure S6: the analysis of neurotoxicity between the two groups. There were no statistically significant differences in neurotoxicity [RR = 0.65, 95% CI (0.35, 1.18), and P = 0.16] between two groups. Figure S7: the analysis of other toxicity between the two groups. Meta-analysis showed that Aidi injection plus docetaxel-based chemotherapy had low risk of alopecia [RR = 0.58, 95% CI (0.36, 0.93), and P = 0.02] using fixed-effects model. But there were [file 7918258.f1.zip › 7918258_SupplDesc.docx]

Meta-analysis results of acute or chronic toxicity (Figure S1-7). Figure S1.The analysis of neutropenia between the two groups. Meta-analysis showed that Aidi injection plus *docetaxel-based* chemotherapy had low risk of neutropenia [RR=0.70, 95%CI(0.61, 0.79), P＜0.00001] using random-effects model. Figure S2.The analysis of thrombocytopenia between the two groups. Meta-analysis showed that Aidi injection plus *docetaxel-based* chemotherapy had low risk of thrombocytopenia [RR=0.63, 95%CI (0.53, 0.75), P＜0.00001] using fixed-effects model. Figure S3.The analysis of anemia between the two groups. Meta-analysis showed that Aidi injection plus *docetaxel-based* chemotherapy had low risk of anemia [RR=0.60, 95%CI(0.48, 0.75) , P＜0.00001] using fixed-effects model. Figure S4.The analysis of gastrointestinal toxicity between the two groups. Meta-analysis showed that Aidi injection plus *docetaxel-based* chemotherapy had low risk of gastrointestinal toxicity [RR=0.76, 95%CI (0.65, 0.89), P=0.0006] using random-effects model. Figure S5.The analysis of hepatorenal dysfunctions between the two groups. Meta-analysis showed that Aidi injection plus *docetaxel-based* chemotherapy had low risk of hepatorenal dysfunctions [RR=0.56, 95%CI (0.36, 0.88), P=0.01] using fixed-effects model. But There were no statistically significant differences in liver dysfunction [RR=0.69, 95%CI(0.47, 1.01), P=0.05], renal dysfunction [RR=0.56, 95%CI (0.31, 1.00), P=0.05] between two groups. Figure S6.The analysis of neurotoxicity between the two groups. There were no statistically significant differences in neurotoxicity [RR=0.65, 95%CI (0.35, 1.18), P=0.16] between two groups. Figure S7.The analysis of other toxicity between the two groups. Meta-analysis showed that Aidi injection plus *docetaxel-based* chemotherapy had low risk of alopecia [RR=0.58, 95%CI(0.36, 0.93), P=0.02] using fixed-effects model. But there were no statistically significant differences in rash [RR=0.75, 95%CI (0.38, 1.49), P=0.42], phlebitis [RR=1.00, 95%CI(0.63, 1.59), P=1.00] and oral mucositis [RR=0.64, 95%CI (0.38, 1.09), P=0.10] between two groups.

Subgroup analysis results of ORR and DCR (Figure S8-13). Figure S8. Subgroup analysis of ORR via drug doses. Subgroup analysis showed that with 100ml, 80-100ml and 50ml, Aidi injection could all increase the ORR. Figure S9. Subgroup analysis of DCR via drug doses. Subgroup analysis showed that with 100ml, 80-100ml and 50ml, Aidi injection could all increase the DCR. Figure S10. Subgroup analysis of ORR via docetaxel-based chemotherapy. Subgroup analysis showed that only Aidi injection plus DP, DC, and DO could increase the DCR. Figure S11. Subgroup analysis of DCR via docetaxel-based chemotherapy. Subgroup analysis showed that only Aidi injection plus DP, DC, and DO could increase the DCR. Figure S12. Subgroup analysis of ORR via evaluation criteria.Subgroup analysis showed that Aidi injection plus *docetaxel-based* chemotherapy could increase the ORR using the WHO or RECIST criteria. Figure S13. Subgroup analysis of DCR via evaluation criteria. Subgroup analysis showed that Aidi injection plus *docetaxel-based* chemotherapy could increase the DCR using the WHO or RECIST criteria.
