# Supplementary material for: Clinical Efficacy and Safety of Aidi Injection Plus Docetaxel-Based Chemotherapy in Advanced Nonsmall Cell Lung Cancer: A Meta-Analysis of 36 Randomized Controlled Trials
Source: Evid Based Complement Alternat Med. 2018 Jun 11;2018:7918258. doi: 10.1155/2018/7918258 (PMC6016159; doi:10.1155/2018/7918258)
Supplement: Supplementary Materials — Meta-analysis results of acute or chronic toxicity (Figures S1-7). Figure S1: the analysis of neutropenia between the two groups. Meta-analysis showed that Aidi injection plus docetaxel-based chemotherapy had low risk of neutropenia [RR = 0.70, 95% CI (0.61, 0.79), and P < 0.00001] using random-effects model. Figure S2: the analysis of thrombocytopenia between the two groups. Meta-analysis showed that Aidi injection plus docetaxel-based chemotherapy had low risk of thrombocytopenia [RR = 0.63, 95% CI (0.53, 0.75), and P < 0.00001] using fixed-effects model. Figure S3: the analysis of anemia between the two groups. Meta-analysis showed that Aidi injection plus docetaxel-based chemotherapy had low risk of anemia [RR = 0.60, 95% CI (0.48, 0.75), and P < 0.00001] using fixed-effects model. Figure S4: the analysis of gastrointestinal toxicity between the two groups. Meta-analysis showed that Aidi injection plus docetaxel-based chemotherapy had low risk of gastrointestinal toxicity [RR = 0.76, 95% CI (0.65, 0.89), and P = 0.0006] using random-effects model. Figure S5: the analysis of hepatorenal dysfunctions between the two groups. Meta-analysis showed that Aidi injection plus docetaxel-based chemotherapy had low risk of hepatorenal dysfunctions [RR = 0.56, 95% CI (0.36, 0.88), and P = 0.01] using fixed-effects model. But there were no statistically significant differences in liver dysfunction [RR = 0.69, 95% CI (0.47, 1.01), and P = 0.05], renal dysfunction [RR = 0.56, 95% CI (0.31, 1.00), and P = 0.05] between two groups. Figure S6: the analysis of neurotoxicity between the two groups. There were no statistically significant differences in neurotoxicity [RR = 0.65, 95% CI (0.35, 1.18), and P = 0.16] between two groups. Figure S7: the analysis of other toxicity between the two groups. Meta-analysis showed that Aidi injection plus docetaxel-based chemotherapy had low risk of alopecia [RR = 0.58, 95% CI (0.36, 0.93), and P = 0.02] using fixed-effects model. But there were [file 7918258.f1.zip › mat.7918258.v3.pdf]

Appendix

Figure S1-7. Meta-analysis results of acute/subacute toxicity

Study or Subgroup	Aidi injection Events	Aidi injection Total	Docetaxel-based chemotherapy Events	Docetaxel-based chemotherapy Total	Weight	Risk Ratio M-H, Random, 95% CI	Year
Zhu,Q 2006	27	30	26	30	6.5%	1.04 [0.86, 1.25]	2006
Bian,M 2006	8	34	14	30	2.3%	0.50 [0.25, 1.03]	2006
Chen,X 2007	11	32	20	32	3.2%	0.55 [0.32, 0.95]	2007
Lin,Q.2008	15	30	23	30	4.3%	0.65 [0.43, 0.98]	2008
Hou,S. 2008	19	35	29	35	5.0%	0.66 [0.47, 0.92]	2008
Jiang, L 2008	47	50	50	50	7.2%	0.94 [0.87, 1.02]	2008
Wang,H 2008	13	40	16	40	3.0%	0.81 [0.45, 1.46]	2008
Lin,S 2011	30	42	36	40	6.2%	0.79 [0.64, 0.99]	2011
Xing, H. 2011	13	35	20	35	3.5%	0.65 [0.39, 1.09]	2011
Jiang,S 2012	11	23	21	23	4.0%	0.52 [0.34, 0.82]	2012
Tang,X 2012	19	36	30	40	4.8%	0.70 [0.49, 1.01]	2012
Ge,C 2013	12	41	19	39	3.1%	0.60 [0.34, 1.07]	2013
Wu,Y 2013	3	19	8	19	1.1%	0.38 [0.12, 1.20]	2013
Xing,G.2014	50	72	56	63	6.5%	0.78 [0.65, 0.93]	2014
Tang, Y.2014	25	47	28	44	4.9%	0.84 [0.59, 1.18]	2014
Song, L.2014	5	32	11	32	1.6%	0.45 [0.18, 1.16]	2014
Li, J.2014	8	25	17	26	2.7%	0.49 [0.26, 0.92]	2014
Xu,H 2014	8	23	15	23	2.7%	0.53 [0.28, 1.01]	2014
Hu,Q.2015	5	35	9	35	1.4%	0.56 [0.21, 1.49]	2015
Gao,Y 2015	16	40	16	48	3.2%	1.20 [0.69, 2.08]	2015
Mo,Y.2015	10	43	21	43	2.8%	0.48 [0.26, 0.89]	2015
Wang,L.2015	49	60	56	60	6.9%	0.88 [0.76, 1.00]	2015
Wang,Y.2016	13	23	19	23	4.4%	0.68 [0.46, 1.03]	2016
He,Z.2016	5	39	12	39	1.5%	0.42 [0.16, 1.07]	2016
Wang,X.2016	14	37	26	36	3.9%	0.52 [0.33, 0.83]	2016
Zhu,J.2017	16	84	29	84	3.4%	0.55 [0.32, 0.94]	2017
**Total (95% CI)**		**1007**		**999**	**100.0%**	**0.70 [0.61, 0.79]**	
Total events	452		627				

Heterogeneity: Tau² = 0.06; Chi² = 93.17, df = 25 (P < 0.00001); I² = 73%
Test for overall effect: Z = 5.40 (P < 0.00001)

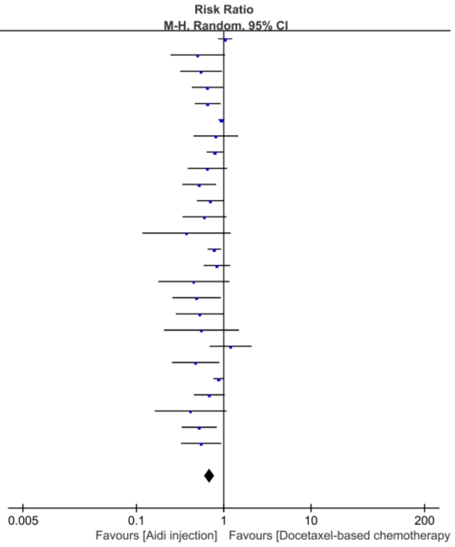

Figure S1.The analysis of neutropenia between the two groups

Study or Subgroup	Aidi injection Events	Aidi injection Total	Docetaxel-based chemotherapy Events	Docetaxel-based chemotherapy Total	Weight	Risk Ratio M-H, Fixed, 95% CI	Year
Zhu,Q 2006	3	30	6	30	2.5%	0.50 [0.14, 1.82]	2006
Jiang, L 2008	18	50	25	50	10.5%	0.72 [0.45, 1.14]	2008
Wang,H 2008	6	40	11	40	4.6%	0.55 [0.22, 1.33]	2008
Lin,S 2011	13	42	20	40	8.6%	0.62 [0.36, 1.07]	2011
Xing, H. 2011	5	35	9	35	3.8%	0.56 [0.21, 1.49]	2011
Jiang,S 2012	4	23	4	23	1.7%	1.00 [0.28, 3.52]	2012
Song, L.2014	4	32	9	32	3.8%	0.44 [0.15, 1.30]	2014
Xu,H 2014	6	23	7	23	2.9%	0.86 [0.34, 2.16]	2014
Tang, Y.2014	19	47	18	44	7.8%	0.99 [0.60, 1.62]	2014
Xing,G.2014	20	72	31	63	13.9%	0.56 [0.36, 0.88]	2014
Mo,Y.2015	2	43	8	43	3.4%	0.25 [0.06, 1.11]	2015
Hu,Q.2015	5	35	11	35	4.6%	0.45 [0.18, 1.17]	2015
Wang,L.2015	11	60	21	60	8.8%	0.52 [0.28, 0.99]	2015
Wang,X.2016	6	37	9	36	3.8%	0.65 [0.26, 1.64]	2016
He,Z.2016	4	39	6	39	2.5%	0.67 [0.20, 2.18]	2016
Wang,Y.2016	14	23	20	23	8.4%	0.70 [0.49, 1.01]	2016
Zhu,J.2017	13	84	20	84	8.4%	0.65 [0.35, 1.22]	2017
**Total (95% CI)**		**715**		**700**	**100.0%**	**0.63 [0.53, 0.75]**	
Total events	153		235				

Heterogeneity: Chi² = 7.91, df = 16 (P = 0.95); I² = 0%
Test for overall effect: Z = 5.36 (P < 0.00001)

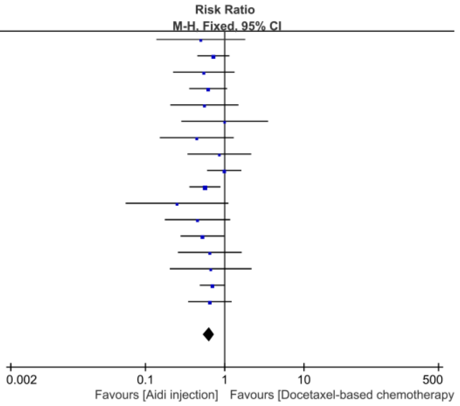

Figure S2.The analysis of thrombocytopenia between the two groups

Study or Subgroup	Aidi injection Events	Aidi injection Total	Docetaxel-based chemotherapy Events	Docetaxel-based chemotherapy Total	Weight	Risk Ratio M-H, Fixed, 95% CI	Year
Zhu,Q 2006	6	30	11	30	8.0%	0.55 [0.23, 1.28]	2006
Jiang, L 2008	15	50	17	50	12.3%	0.88 [0.50, 1.57]	2008
Xing, H. 2011	4	35	11	35	8.0%	0.36 [0.13, 1.03]	2011
Jiang,S 2012	2	23	3	23	2.2%	0.67 [0.12, 3.62]	2012
Xu,H 2014	7	23	15	23	10.9%	0.47 [0.23, 0.93]	2014
Xing,G.2014	25	72	39	63	30.2%	0.56 [0.39, 0.81]	2014
Wang,L.2015	13	60	14	60	10.2%	0.93 [0.48, 1.81]	2015
Wang,Y.2016	3	23	6	23	4.4%	0.50 [0.14, 1.76]	2016
Wang,X.2016	10	37	19	36	14.0%	0.51 [0.28, 0.95]	2016
**Total (95% CI)**		**353**		**343**	**100.0%**	**0.60 [0.48, 0.75]**	
Total events	85		135				

Heterogeneity: Chi² = 5.32, df = 8 (P = 0.72); I² = 0%
Test for overall effect: Z = 4.48 (P < 0.00001)

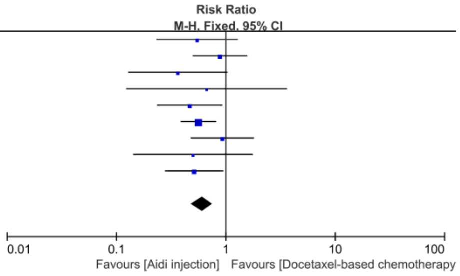

Figure S3.The analysis of anemia between the two groups

Study or Subgroup	Aidi injection Events	Aidi injection Total	Docetaxel-based chemotherapy Events	Docetaxel-based chemotherapy Total	Weight	Risk Ratio M-H, Random, 95% CI	Year
Zhu,Q 2006	19	30	26	30	4.9%	0.73 [0.54, 0.99]	2006
Bian,M 2006	10	34	11	30	2.8%	0.80 [0.40, 1.62]	2006
Chen,X 2007	11	32	14	32	3.2%	0.79 [0.42, 1.46]	2007
Hou,S. 2008	13	35	15	35	3.4%	0.87 [0.49, 1.54]	2008
Lin,Q.2008	18	30	17	30	4.2%	1.06 [0.69, 1.62]	2008
Wang,H 2008	10	40	14	40	2.9%	0.71 [0.36, 1.41]	2008
Jiang, L 2008	50	50	50	50	6.0%	1.00 [0.96, 1.04]	2008
Lin,S 2011	37	42	36	40	5.7%	0.98 [0.84, 1.14]	2011
Du,Z 2011	29	60	43	60	4.9%	0.67 [0.50, 0.92]	2011
Wang,T 2011	18	49	40	49	4.4%	0.45 [0.30, 0.67]	2011
Xing, H. 2011	6	35	15	35	2.3%	0.40 [0.18, 0.91]	2011
Jiang,S 2012	6	23	8	23	2.1%	0.75 [0.31, 1.82]	2012
Tang,X 2012	17	36	28	40	4.4%	0.67 [0.45, 1.01]	2012
Ge,C 2013	14	41	12	39	3.1%	1.11 [0.59, 2.09]	2013
Tang, Y.2014	28	47	32	44	5.0%	0.82 [0.61, 1.10]	2014
Li, J.2014	5	25	12	26	2.1%	0.43 [0.18, 1.05]	2014
Xu,H 2014	7	23	17	23	2.9%	0.41 [0.21, 0.80]	2014
Song, L.2014	11	32	14	32	3.2%	0.79 [0.42, 1.46]	2014
Xing,G.2014	63	72	56	63	5.8%	0.98 [0.87, 1.11]	2014
Wang,L.2015	52	60	54	60	5.8%	0.96 [0.85, 1.10]	2015
Hu,Q.2015	11	35	14	35	3.1%	0.79 [0.42, 1.48]	2015
Gao,Y 2015	15	40	18	48	3.5%	1.00 [0.58, 1.72]	2015
Mo,Y.2015	13	43	22	43	3.6%	0.59 [0.34, 1.01]	2015
He,Z.2016	12	39	15	39	3.2%	0.80 [0.43, 1.48]	2016
Wang,Y.2016	11	23	18	23	3.9%	0.61 [0.38, 0.99]	2016
Zhu,J.2017	18	84	33	84	3.8%	0.55 [0.33, 0.89]	2017
**Total (95% CI)**		**1060**		**1053**	**100.0%**	**0.76 [0.65, 0.89]**	
Total events	504		634				

Heterogeneity: Tau² = 0.11; Chi² = 209.56, df = 25 (P < 0.00001); I² = 88%
Test for overall effect: Z = 3.42 (P = 0.0006)

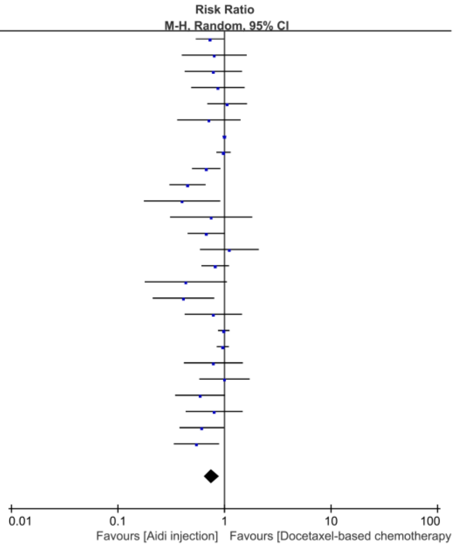

Figure S4.The analysis of gastrointestinal toxicity between the two groups

Study or Subgroup	Aidi injection Events	Aidi injection Total	Docetaxel-based chemotherapy Events	Docetaxel-based chemotherapy Total	Weight	Risk Ratio M-H, Fixed, 95% CI	Year
**Liver dysfunction**							
Bian,M 2006	2	34	2	30	4.0%	0.88 [0.13, 5.88]	2006
Wang,H 2008	4	40	7	40	13.3%	0.57 [0.18, 1.80]	2008
Lin,S 2011	4	42	5	40	9.7%	0.76 [0.22, 2.64]	2011
Tang,X 2012	5	36	11	40	19.8%	0.51 [0.19, 1.31]	2012
Xing,G.2014	6	72	8	63	16.2%	0.66 [0.24, 1.79]	2014
Tang, Y.2014	13	47	15	44	29.4%	0.81 [0.44, 1.51]	2014
Wang,X.2016	3	37	4	36	7.7%	0.73 [0.18, 3.03]	2016
**Subtotal (95% CI)**		**308**		**293**	**100.0%**	**0.69 [0.47, 1.01]**	
Total events	37		52				
Heterogeneity: Chi² = 0.88, df = 6 (P = 0.99); I² = 0%							
Test for overall effect: Z = 1.93 (P = 0.05)							
**Renal dysfunction**							
Bian,M 2006	0	34	1	30	5.9%	0.30 [0.01, 6.99]	2006
Wang,H 2008	2	40	4	40	14.8%	0.50 [0.10, 2.58]	2008
Tang, Y.2014	9	47	11	44	42.0%	0.77 [0.35, 1.67]	2014
Wang,X.2016	2	37	6	36	22.5%	0.32 [0.07, 1.50]	2016
Wang,Y.2016	2	23	4	23	14.8%	0.50 [0.10, 2.47]	2016
**Subtotal (95% CI)**		**181**		**173**	**100.0%**	**0.56 [0.31, 1.00]**	
Total events	15		26				
Heterogeneity: Chi² = 1.30, df = 4 (P = 0.86); I² = 0%							
Test for overall effect: Z = 1.96 (P = 0.05)							
**Hepatorenal dysfunctions**							
Xing, H. 2011	1	35	5	35	12.4%	0.20 [0.02, 1.63]	2011
Jiang,S 2012	5	23	7	23	17.3%	0.71 [0.27, 1.93]	2012
Ge,C 2013	10	41	17	39	43.2%	0.56 [0.29, 1.07]	2013
Li, J.2014	2	25	4	26	9.7%	0.52 [0.10, 2.59]	2014
Xu,H 2014	5	23	7	23	17.3%	0.71 [0.27, 1.93]	2014
**Subtotal (95% CI)**		**147**		**146**	**100.0%**	**0.56 [0.36, 0.88]**	
Total events	23		40				
Heterogeneity: Chi² = 1.38, df = 4 (P = 0.85); I² = 0%							
Test for overall effect: Z = 2.51 (P = 0.01)							

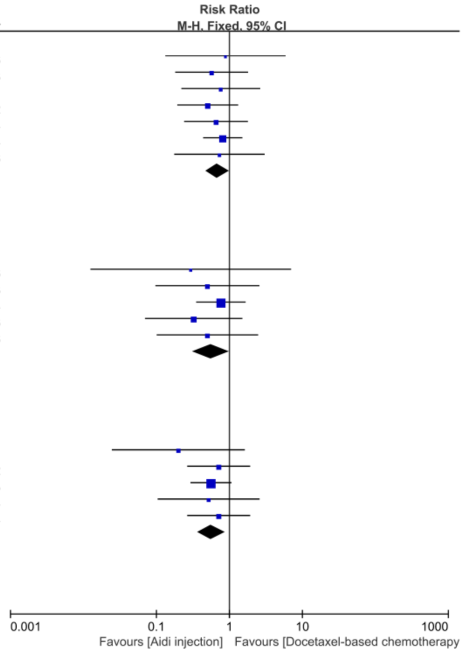

Figure S5.The analysis of hepatorenal dysfunctions between the two groups

Study or Subgroup	Aidi injection Events	Aidi injection Total	Docetaxel-based chemotherapy Events	Docetaxel-based chemotherapy Total	Weight	Risk Ratio M-H, Random, 95% CI	Year
Bian,M 2006	4	34	4	30	14.3%	0.88 [0.24, 3.22]	2006
Xing, H. 2011	1	35	8	35	7.3%	0.13 [0.02, 0.95]	2011
Wang,T 2011	16	49	35	49	34.3%	0.46 [0.29, 0.71]	2011
Li, J.2014	3	25	4	26	13.0%	0.78 [0.19, 3.14]	2014
Tang, Y.2014	18	47	15	44	31.1%	1.12 [0.65, 1.94]	2014
**Total (95% CI)**		**190**		**184**	**100.0%**	**0.65 [0.35, 1.18]**	
Total events	42		66				

Heterogeneity: Tau² = 0.23; Chi² = 9.16, df = 4 (P = 0.06); I² = 56%
Test for overall effect: Z = 1.41 (P = 0.16)

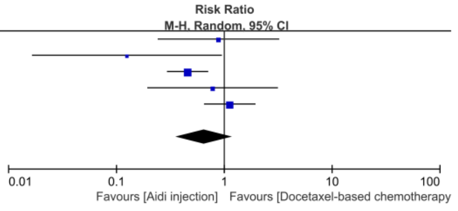

Figure S6.The analysis of neurotoxicity between the two groups

Study or Subgroup	Aidi injection Events	Aidi injection Total	Docetaxel-based chemotherapy Events	Docetaxel-based chemotherapy Total	Weight	Risk Ratio M-H, Fixed, 95% CI	Year
**Alopecia**							
Bian,M 2006	3	34	3	30	11.7%	0.88 [0.19, 4.05]	2006
Ge,C 2013	3	41	6	39	22.5%	0.48 [0.13, 1.77]	2013
Xu,H 2014	10	23	18	23	65.8%	0.56 [0.33, 0.93]	2014
**Subtotal (95% CI)**		**98**		**92**	**100.0%**	**0.58 [0.36, 0.93]**	
Total events	16		27				
Heterogeneity: Chi² = 0.40, df = 2 (P = 0.82); I² = 0%							
Test for overall effect: Z = 2.25 (P = 0.02)							
**Rash**							
Ge,C 2013	2	41	5	39	33.2%	0.38 [0.08, 1.85]	2013
Tang, Y.2014	10	47	10	44	66.8%	0.94 [0.43, 2.03]	2014
**Subtotal (95% CI)**		**88**		**83**	**100.0%**	**0.75 [0.38, 1.49]**	
Total events	12		15				
Heterogeneity: Chi² = 1.02, df = 1 (P = 0.31); I² = 2%							
Test for overall effect: Z = 0.81 (P = 0.42)							
**Phlebitis**							
Zhu,Q 2006	1	30	0	30	2.0%	3.00 [0.13, 70.83]	2006
Jiang,S 2012	7	23	9	23	35.3%	0.78 [0.35, 1.73]	2012
Wang,L.2015	17	60	16	60	62.7%	1.06 [0.59, 1.90]	2015
**Subtotal (95% CI)**		**113**		**113**	**100.0%**	**1.00 [0.63, 1.59]**	
Total events	25		25				
Heterogeneity: Chi² = 0.88, df = 2 (P = 0.64); I² = 0%							
Test for overall effect: Z = 0.00 (P = 1.00)							
**Oral mucositis**							
Song, L.2014	6	32	7	32	25.0%	0.86 [0.32, 2.27]	2014
Hu,Q.2015	6	35	7	35	25.0%	0.86 [0.32, 2.29]	2015
Mo,Y.2015	6	43	14	43	50.0%	0.43 [0.18, 1.01]	2015
**Subtotal (95% CI)**		**110**		**110**	**100.0%**	**0.64 [0.38, 1.09]**	
Total events	18		28				
Heterogeneity: Chi² = 1.52, df = 2 (P = 0.47); I² = 0%							
Test for overall effect: Z = 1.63 (P = 0.10)							

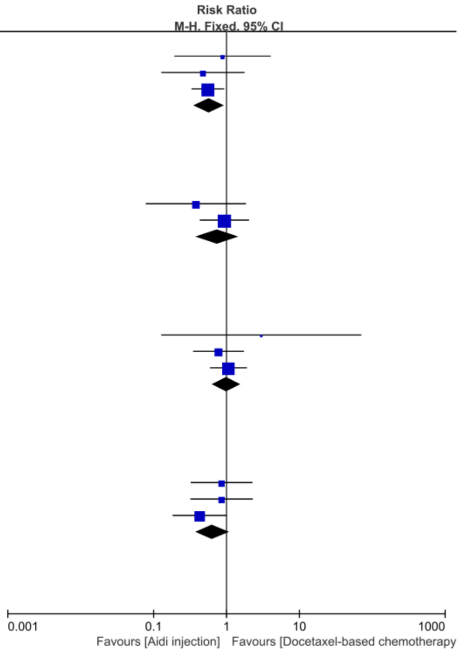

Figure S7.The analysis of other toxicity between the two groups

Figure S8-13. Subgroups analysis of ORR and DCR

Study or Subgroup	Aidi injection Events	Aidi injection Total	Docetaxel-based chemotherapy Events	Docetaxel-based chemotherapy Total	Weight	Risk Ratio M-H, Fixed, 95% CI	Year
**Aidi injection(100ml)**							
Shi,L 2012	22	38	16	38	3.1%	1.38 [0.87, 2.18]	2012
Xing,G.2014	42	72	26	63	5.3%	1.41 [0.99, 2.01]	2014
Li, J.2014	13	25	10	26	1.9%	1.35 [0.73, 2.50]	2014
**Subtotal (95% CI)**		**135**		**127**	**10.3%**	**1.39 [1.08, 1.80]**	
Total events	77		52				
Heterogeneity: Chi² = 0.02, df = 2 (P = 0.99); I² = 0%							
Test for overall effect: Z = 2.53 (P = 0.01)							
**Aidi injection(80-100ml)**							
Wang,H 2008	23	40	16	40	3.1%	1.44 [0.90, 2.29]	2008
Cui, H.2010	19	30	11	30	2.1%	1.73 [1.00, 2.97]	2010
Wang,T 2011	35	49	28	49	5.4%	1.25 [0.93, 1.69]	2011
Wang,J 2015	30	50	23	50	4.4%	1.30 [0.90, 1.90]	2015
**Subtotal (95% CI)**		**169**		**169**	**15.0%**	**1.37 [1.13, 1.67]**	
Total events	107		78				
Heterogeneity: Chi² = 1.17, df = 3 (P = 0.76); I² = 0%							
Test for overall effect: Z = 3.13 (P = 0.002)							
**Aidi injection(80ml)**							
Gao,Y 2015	20	40	20	48	3.5%	1.20 [0.76, 1.89]	2015
Gao,Y.2016	26	50	16	50	3.1%	1.63 [1.00, 2.64]	2016
**Subtotal (95% CI)**		**90**		**98**	**6.6%**	**1.40 [1.00, 1.95]**	
Total events	46		36				
Heterogeneity: Chi² = 0.80, df = 1 (P = 0.37); I² = 0%							
Test for overall effect: Z = 1.98 (P = 0.05)							
**Aidi injection(60ml)**							
Wu,Y 2013	5	19	4	19	0.8%	1.25 [0.40, 3.95]	2013
**Subtotal (95% CI)**		**19**		**19**	**0.8%**	**1.25 [0.40, 3.95]**	
Total events	5		4				
Heterogeneity: Not applicable							
Test for overall effect: Z = 0.38 (P = 0.70)							
**Aidi injection(50ml)**							
Zhu,Q 2006	16	30	14	30	2.7%	1.14 [0.69, 1.90]	2006
Bian,M 2006	20	34	10	30	2.0%	1.76 [0.99, 3.15]	2006
Chen,X 2007	17	32	13	32	2.5%	1.31 [0.77, 2.22]	2007
Hou,E. 2008	19	35	16	35	3.1%	1.19 [0.74, 1.90]	2008
Jiang, L 2008	18	50	16	50	3.1%	1.13 [0.65, 1.95]	2008
Lin,Q.2008	14	30	12	30	2.3%	1.17 [0.65, 2.09]	2008
Tang,L 2011	10	25	9	25	1.7%	1.11 [0.55, 2.26]	2011
Lin,S 2011	17	42	15	40	3.0%	1.08 [0.63, 1.86]	2011
Xing, H. 2011	14	35	11	35	2.1%	1.27 [0.67, 2.40]	2011
Tang,X 2012	20	36	13	40	2.4%	1.71 [1.00, 2.91]	2012
Jiang,S 2012	11	23	9	23	1.7%	1.22 [0.63, 2.38]	2012
Ge,C 2013	9	41	4	39	0.8%	2.14 [0.72, 6.39]	2013
Chen,Z 2013	25	54	21	52	4.1%	1.15 [0.74, 1.78]	2013
Gao,E 2014	14	36	15	35	2.9%	0.91 [0.52, 1.59]	2014
Song, L.2014	10	32	9	32	1.7%	1.11 [0.52, 2.37]	2014
Xu,H 2014	9	23	7	23	1.3%	1.29 [0.58, 2.86]	2014
Tang, Y.2014	34	47	23	44	4.6%	1.38 [0.99, 1.93]	2014
Mo,Y.2015	23	43	18	43	3.5%	1.28 [0.82, 2.00]	2015
Hu,Q.2015	11	35	10	35	1.9%	1.10 [0.54, 2.25]	2015
Wang,L.2015	24	60	21	60	4.0%	1.14 [0.72, 1.82]	2015
Wang,Y.2016	14	23	12	23	2.3%	1.17 [0.70, 1.94]	2016
Zhu,J.2017	48	84	30	84	5.8%	1.60 [1.14, 2.25]	2017
**Subtotal (95% CI)**		**850**		**840**	**59.6%**	**1.27 [1.14, 1.42]**	
Total events	397		308				
Heterogeneity: Chi² = 8.51, df = 21 (P = 0.99); I² = 0%							
Test for overall effect: Z = 4.21 (P < 0.0001)							
**Aidi injection(40ml)**							
Du,Z 2011	33	60	28	60	5.4%	1.18 [0.83, 1.68]	2011
**Subtotal (95% CI)**		**60**		**60**	**5.4%**	**1.18 [0.83, 1.68]**	
Total events	33		28				
Heterogeneity: Not applicable							
Test for overall effect: Z = 0.91 (P = 0.36)							
**Aidi injection(Unclear)**							
He,Z.2016	14	39	12	39	2.3%	1.17 [0.62, 2.19]	2016
**Subtotal (95% CI)**		**39**		**39**	**2.3%**	**1.17 [0.62, 2.19]**	
Total events	14		12				
Heterogeneity: Not applicable							
Test for overall effect: Z = 0.48 (P = 0.63)							
**Total (95% CI)**		**1362**		**1352**	**100.0%**	**1.30 [1.19, 1.42]**	
Total events	679		518				
Heterogeneity: Chi² = 11.58, df = 33 (P = 1.00); I² = 0%							
Test for overall effect: Z = 6.08 (P < 0.00001)							
Test for subgroup differences: Chi² = 1.28, df = 6 (P = 0.97), I² = 0%							

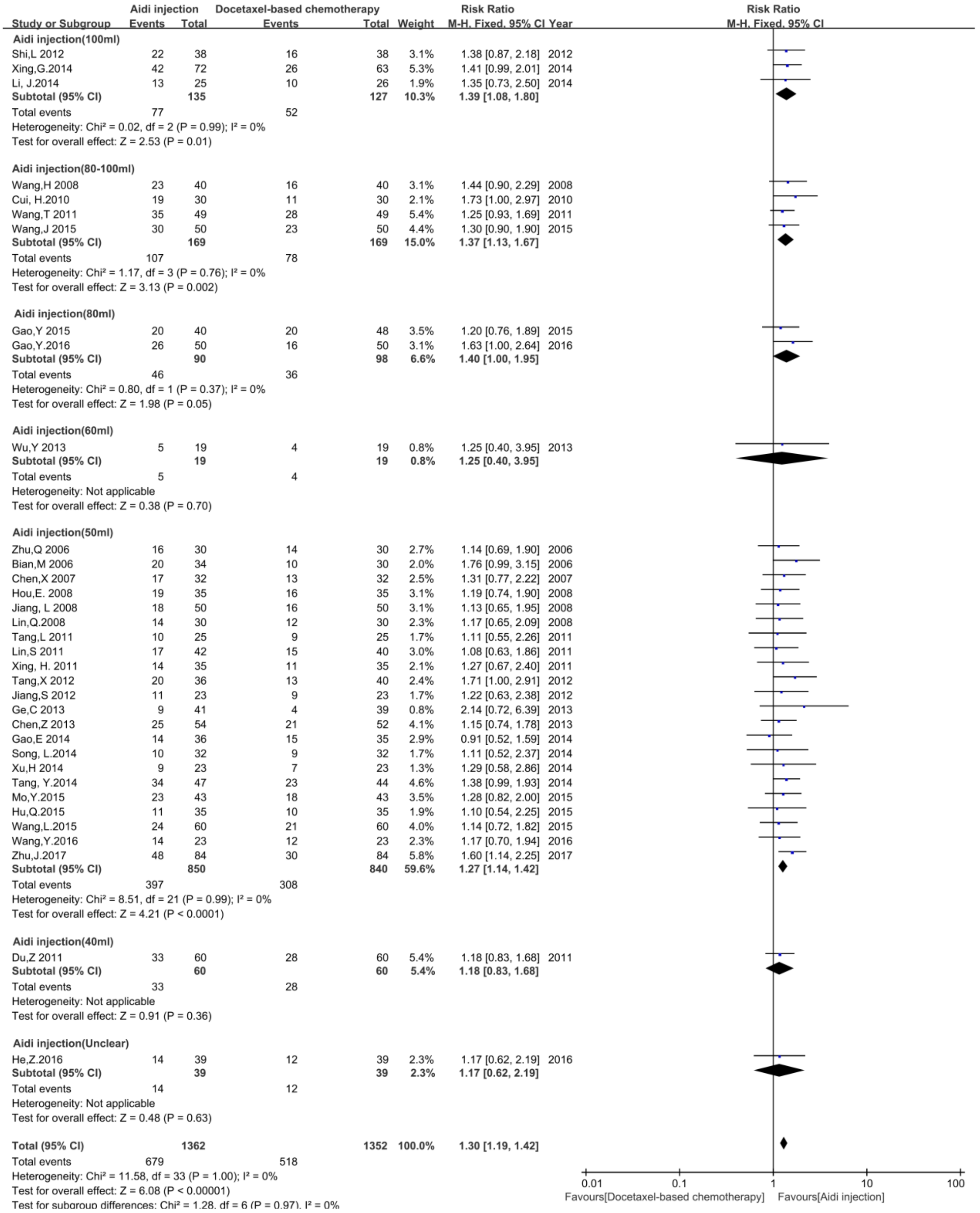

Figure S8. Subgroup analysis of ORR via drug doses

Study or Subgroup	Aidi injection Events	Aidi injection Total	Docetaxel-based chemotherapy Events	Docetaxel-based chemotherapy Total	Weight	Risk Ratio M-H, Fixed, 95% CI	Year
**Aidi injection(100ml)**							
Shi,L 2012	32	38	27	38	2.9%	1.19 [0.93, 1.51]	2012
Xing,G.2014	52	72	31	63	3.5%	1.47 [1.10, 1.96]	2014
Li, J.2014	19	25	19	26	2.0%	1.04 [0.75, 1.43]	2014
**Subtotal (95% CI)**		**135**		**127**	**8.4%**	**1.27 [1.07, 1.50]**	
Total events	103		77				
Heterogeneity: Chi² = 2.75, df = 2 (P = 0.25); I² = 27%							
Test for overall effect: Z = 2.78 (P = 0.005)							
**Aidi injection(80-100ml)**							
Wang,H 2008	31	40	23	40	2.5%	1.35 [0.98, 1.85]	2008
Cui, H.2010	28	30	26	30	2.8%	1.08 [0.91, 1.28]	2010
Wang,T 2011	45	49	38	49	4.1%	1.18 [1.00, 1.41]	2011
Wang,J 2015	39	50	33	50	3.5%	1.18 [0.92, 1.51]	2015
**Subtotal (95% CI)**		**169**		**169**	**12.8%**	**1.19 [1.06, 1.33]**	
Total events	143		120				
Heterogeneity: Chi² = 1.96, df = 3 (P = 0.58); I² = 0%							
Test for overall effect: Z = 3.03 (P = 0.002)							
**Aidi injection(80ml)**							
Gao,Y 2015	28	40	29	48	2.8%	1.16 [0.85, 1.57]	2015
Gao,Y.2016	36	50	27	50	2.9%	1.33 [0.98, 1.82]	2016
**Subtotal (95% CI)**		**90**		**98**	**5.7%**	**1.25 [1.00, 1.55]**	
Total events	64		56				
Heterogeneity: Chi² = 0.40, df = 1 (P = 0.53); I² = 0%							
Test for overall effect: Z = 1.99 (P = 0.05)							
**Aidi injection(60ml)**							
Wu,Y 2013	7	19	6	19	0.6%	1.17 [0.48, 2.83]	2013
**Subtotal (95% CI)**		**19**		**19**	**0.6%**	**1.17 [0.48, 2.83]**	
Total events	7		6				
Heterogeneity: Not applicable							
Test for overall effect: Z = 0.34 (P = 0.73)							
**Aidi injection(50ml)**							
Bian,M 2006	27	34	21	30	2.4%	1.13 [0.85, 1.52]	2006
Zhu,Q 2006	29	30	28	30	3.0%	1.04 [0.92, 1.16]	2006
Chen,X 2007	28	32	22	32	2.3%	1.27 [0.97, 1.66]	2007
Jiang, L 2008	46	50	40	50	4.3%	1.15 [0.98, 1.35]	2008
Hou,E. 2008	30	35	28	35	3.0%	1.07 [0.87, 1.33]	2008
Lin,Q.2008	28	30	22	30	2.3%	1.27 [1.01, 1.61]	2008
Xing, H. 2011	27	35	25	35	2.7%	1.08 [0.82, 1.42]	2011
Lin,S 2011	39	42	31	40	3.4%	1.20 [0.99, 1.44]	2011
Tang,X 2012	30	36	29	40	2.9%	1.15 [0.90, 1.46]	2012
Jiang,S 2012	22	23	17	23	1.8%	1.29 [1.00, 1.67]	2012
Chen,Z 2013	35	54	25	52	2.7%	1.35 [0.96, 1.90]	2013
Ge,C 2013	23	41	13	39	1.4%	1.68 [1.00, 2.83]	2013
Gao,E 2014	31	36	29	35	3.1%	1.04 [0.85, 1.27]	2014
Xu,H 2014	18	23	17	23	1.8%	1.06 [0.77, 1.46]	2014
Tang, Y.2014	43	47	37	44	4.1%	1.09 [0.93, 1.27]	2014
Song, L.2014	30	32	27	32	2.9%	1.11 [0.93, 1.32]	2014
Mo,Y.2015	36	43	28	43	3.0%	1.29 [1.00, 1.66]	2015
Wang,L.2015	44	60	42	60	4.5%	1.05 [0.84, 1.31]	2015
Hu,Q.2015	33	35	30	35	3.2%	1.10 [0.94, 1.29]	2015
Wang,Y.2016	22	23	21	23	2.2%	1.05 [0.90, 1.22]	2016
Zhu,J.2017	77	84	65	84	6.9%	1.18 [1.04, 1.35]	2017
**Subtotal (95% CI)**		**825**		**815**	**64.0%**	**1.16 [1.10, 1.21]**	
Total events	698		597				
Heterogeneity: Chi² = 14.64, df = 20 (P = 0.80); I² = 0%							
Test for overall effect: Z = 5.84 (P < 0.00001)							
**Aidi injection(40ml)**							
Du,Z 2011	53	60	50	60	5.3%	1.06 [0.92, 1.23]	2011
**Subtotal (95% CI)**		**60**		**60**	**5.3%**	**1.06 [0.92, 1.23]**	
Total events	53		50				
Heterogeneity: Not applicable							
Test for overall effect: Z = 0.78 (P = 0.43)							
**Aidi injection(Unclear)**							
He,Z.2016	36	39	30	39	3.2%	1.20 [0.99, 1.46]	2016
**Subtotal (95% CI)**		**39**		**39**	**3.2%**	**1.20 [0.99, 1.46]**	
Total events	36		30				
Heterogeneity: Not applicable							
Test for overall effect: Z = 1.84 (P = 0.07)							
**Total (95% CI)**		**1337**		**1327**	**100.0%**	**1.17 [1.12, 1.22]**	
Total events	1104		936				
Heterogeneity: Chi² = 23.73, df = 32 (P = 0.85); I² = 0%							
Test for overall effect: Z = 7.59 (P < 0.00001)							
Test for subgroup differences: Chi² = 3.31, df = 6 (P = 0.77), I² = 0%							

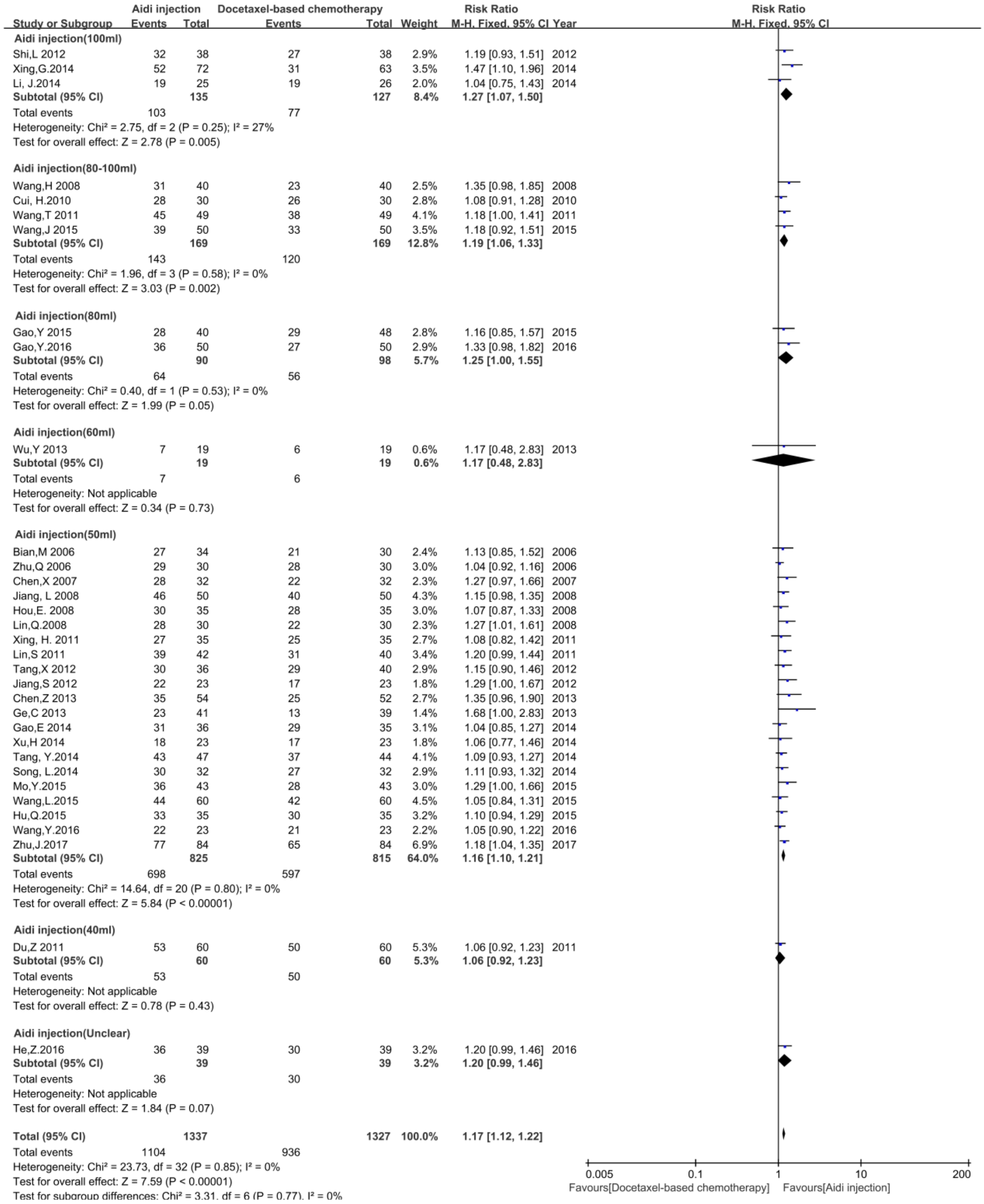

Figure S9. Subgroup analysis of DCR via drug doses

Study or Subgroup	Aidi injection Events	Aidi injection Total	Docetaxel-based chemotherapy Events	Docetaxel-based chemotherapy Total	Weight	Risk Ratio M-H, Fixed, 95% CI	Year
**Aidi injection plus DP VS DP**							
Bian,M 2006	20	34	10	30	2.0%	1.76 [0.99, 3.15]	2006
Zhu,Q 2006	16	30	14	30	2.7%	1.14 [0.69, 1.90]	2006
Chen,X 2007	17	32	13	32	2.5%	1.31 [0.77, 2.22]	2007
Hou,E. 2008	19	35	16	35	3.1%	1.19 [0.74, 1.90]	2008
Lin,Q.2008	14	30	12	30	2.3%	1.17 [0.65, 2.09]	2008
Wang,H 2008	23	40	16	40	3.1%	1.44 [0.90, 2.29]	2008
Jiang, L 2008	18	50	16	50	3.1%	1.13 [0.65, 1.95]	2008
Cui, H.2010	19	30	11	30	2.1%	1.73 [1.00, 2.97]	2010
Wang,T 2011	35	49	28	49	5.4%	1.25 [0.93, 1.69]	2011
Lin,S 2011	17	42	15	40	3.0%	1.08 [0.63, 1.86]	2011
Tang,L 2011	10	25	9	25	1.7%	1.11 [0.55, 2.26]	2011
Du,Z 2011	33	60	28	60	5.4%	1.18 [0.83, 1.68]	2011
Shi,L 2012	22	38	16	38	3.1%	1.38 [0.87, 2.18]	2012
Jiang,S 2012	11	23	9	23	1.7%	1.22 [0.63, 2.38]	2012
Tang,X 2012	20	36	13	40	2.4%	1.71 [1.00, 2.91]	2012
Ge,C 2013	9	41	4	39	0.8%	2.14 [0.72, 6.39]	2013
Chen,Z 2013	25	54	21	52	4.1%	1.15 [0.74, 1.78]	2013
Wu,Y 2013	5	19	4	19	0.8%	1.25 [0.40, 3.95]	2013
Gao,E 2014	14	36	15	35	2.9%	0.91 [0.52, 1.59]	2014
Xu,H 2014	9	23	7	23	1.3%	1.29 [0.58, 2.86]	2014
Gao,Y 2015	20	40	20	48	3.5%	1.20 [0.76, 1.89]	2015
Wang,J 2015	30	50	23	50	4.4%	1.30 [0.90, 1.90]	2015
Mo,Y.2015	23	43	18	43	3.5%	1.28 [0.82, 2.00]	2015
Wang,Y.2016	14	23	12	23	2.3%	1.17 [0.70, 1.94]	2016
**Subtotal (95% CI)**		**883**		**884**	**67.2%**	**1.27 [1.15, 1.41]**	
Total events	443		350				
Heterogeneity: Chi² = 7.90, df = 23 (P = 1.00); I² = 0%							
Test for overall effect: Z = 4.54 (P < 0.00001)							
**Aidi injection plus DC VS DC**							
Song, L.2014	10	32	9	32	1.7%	1.11 [0.52, 2.37]	2014
Hu,Q.2015	11	35	10	35	1.9%	1.10 [0.54, 2.25]	2015
He,Z.2016	14	39	12	39	2.3%	1.17 [0.62, 2.19]	2016
Zhu,J.2017	48	84	30	84	5.8%	1.60 [1.14, 2.25]	2017
**Subtotal (95% CI)**		**190**		**190**	**11.7%**	**1.36 [1.05, 1.76]**	
Total events	83		61				
Heterogeneity: Chi² = 1.71, df = 3 (P = 0.64); I² = 0%							
Test for overall effect: Z = 2.32 (P = 0.02)							
**Aidi injection plus DO VS DO**							
Li, J.2014	13	25	10	26	1.9%	1.35 [0.73, 2.50]	2014
Tang, Y.2014	34	47	23	44	4.6%	1.38 [0.99, 1.93]	2014
**Subtotal (95% CI)**		**72**		**70**	**6.5%**	**1.37 [1.02, 1.85]**	
Total events	47		33				
Heterogeneity: Chi² = 0.00, df = 1 (P = 0.95); I² = 0%							
Test for overall effect: Z = 2.11 (P = 0.04)							
**Aidi injection plus DL VS DL**							
Xing,G.2014	42	72	26	63	5.3%	1.41 [0.99, 2.01]	2014
**Subtotal (95% CI)**		**72**		**63**	**5.3%**	**1.41 [0.99, 2.01]**	
Total events	42		26				
Heterogeneity: Not applicable							
Test for overall effect: Z = 1.92 (P = 0.05)							
**Aidi injection plus DN VS DN**							
Gao,Y.2016	26	50	16	50	3.1%	1.63 [1.00, 2.64]	2016
**Subtotal (95% CI)**		**50**		**50**	**3.1%**	**1.63 [1.00, 2.64]**	
Total events	26		16				
Heterogeneity: Not applicable							
Test for overall effect: Z = 1.97 (P = 0.05)							
**Aidi injection plus docetaxel VS Docetaxel**							
Xing, H. 2011	14	35	11	35	2.1%	1.27 [0.67, 2.40]	2011
Wang,L.2015	24	60	21	60	4.0%	1.14 [0.72, 1.82]	2015
**Subtotal (95% CI)**		**95**		**95**	**6.2%**	**1.19 [0.82, 1.73]**	
Total events	38		32				
Heterogeneity: Chi² = 0.07, df = 1 (P = 0.79); I² = 0%							
Test for overall effect: Z = 0.90 (P = 0.37)							
**Total (95% CI)**		**1362**		**1352**	**100.0%**	**1.30 [1.19, 1.42]**	
Total events	679		518				
Heterogeneity: Chi² = 11.58, df = 33 (P = 1.00); I² = 0%							
Test for overall effect: Z = 6.08 (P < 0.00001)							
Test for subgroup differences: Chi² = 1.71, df = 5 (P = 0.89), I² = 0%							

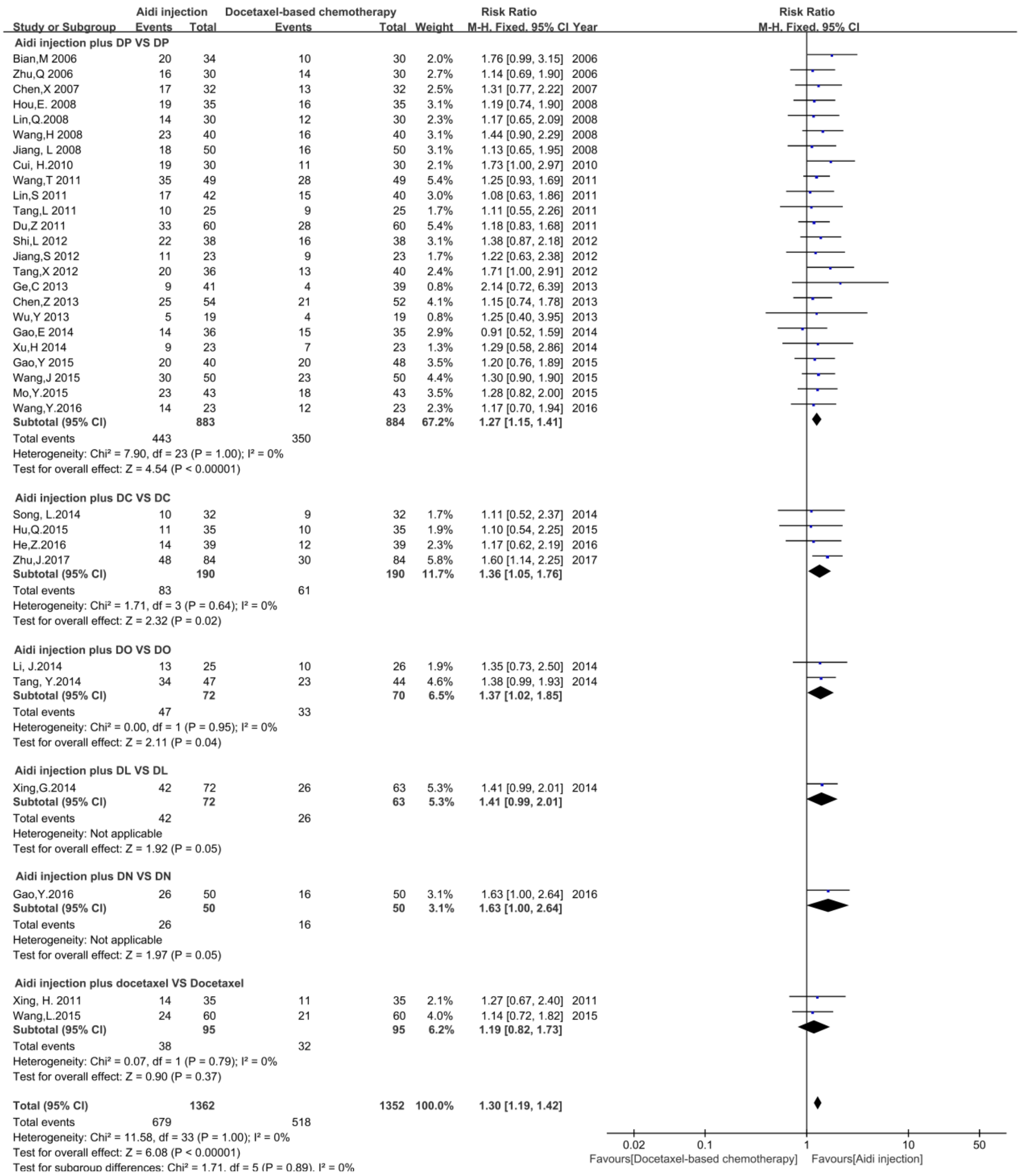

Figure S10. Subgroup analysis of ORR via docetaxel-based chemotherapy

Study or Subgroup	Aidi injection Events	Aidi injection Total	Docetaxel-based chemotherapy Events	Docetaxel-based chemotherapy Total	Weight	Risk Ratio M-H, Fixed, 95% CI	Year
**Aidi injection plus DP VS DP**							
Bian,M 2006	27	34	21	30	2.4%	1.13 [0.85, 1.52]	2006
Zhu,Q 2006	29	30	28	30	3.0%	1.04 [0.92, 1.16]	2006
Chen,X 2007	28	32	22	32	2.3%	1.27 [0.97, 1.66]	2007
Jiang, L 2008	46	50	40	50	4.3%	1.15 [0.98, 1.35]	2008
Lin,Q.2008	28	30	22	30	2.3%	1.27 [1.01, 1.61]	2008
Wang,H 2008	31	40	23	40	2.5%	1.35 [0.98, 1.85]	2008
Hou,E. 2008	30	35	28	35	3.0%	1.07 [0.87, 1.33]	2008
Cui, H.2010	28	30	26	30	2.8%	1.08 [0.91, 1.28]	2010
Wang,T 2011	45	49	38	49	4.1%	1.18 [1.00, 1.41]	2011
Lin,S 2011	39	42	31	40	3.4%	1.20 [0.99, 1.44]	2011
Du,Z 2011	53	60	50	60	5.3%	1.06 [0.92, 1.23]	2011
Tang,X 2012	30	36	29	40	2.9%	1.15 [0.90, 1.46]	2012
Jiang,S 2012	22	23	17	23	1.8%	1.29 [1.00, 1.67]	2012
Shi,L 2012	32	38	27	38	2.9%	1.19 [0.93, 1.51]	2012
Chen,Z 2013	35	54	25	52	2.7%	1.35 [0.96, 1.90]	2013
Ge,C 2013	23	41	13	39	1.4%	1.68 [1.00, 2.83]	2013
Wu,Y 2013	7	19	6	19	0.6%	1.17 [0.48, 2.83]	2013
Xu,H 2014	18	23	17	23	1.8%	1.06 [0.77, 1.46]	2014
Gao,E 2014	31	36	29	35	3.1%	1.04 [0.85, 1.27]	2014
Wang,J 2015	39	50	33	50	3.5%	1.18 [0.92, 1.51]	2015
Mo,Y.2015	36	43	28	43	3.0%	1.29 [1.00, 1.66]	2015
Gao,Y 2015	28	40	29	48	2.8%	1.16 [0.85, 1.57]	2015
Wang,Y.2016	22	23	21	23	2.2%	1.05 [0.90, 1.22]	2016
**Subtotal (95% CI)**		**858**		**859**	**64.2%**	**1.17 [1.12, 1.24]**	
Total events	707		603				
Heterogeneity: Chi² = 17.21, df = 22 (P = 0.75); I² = 0%							
Test for overall effect: Z = 6.16 (P < 0.00001)							
**Aidi injection plus DC VS DC**							
Song, L.2014	30	32	27	32	2.9%	1.11 [0.93, 1.32]	2014
Hu,Q.2015	33	35	30	35	3.2%	1.10 [0.94, 1.29]	2015
He,Z.2016	36	39	30	39	3.2%	1.20 [0.99, 1.46]	2016
Zhu,J.2017	77	84	65	84	6.9%	1.18 [1.04, 1.35]	2017
**Subtotal (95% CI)**		**190**		**190**	**16.2%**	**1.16 [1.07, 1.26]**	
Total events	176		152				
Heterogeneity: Chi² = 0.87, df = 3 (P = 0.83); I² = 0%							
Test for overall effect: Z = 3.53 (P = 0.0004)							
**Aidi injection plus DO VS DO**							
Li, J.2014	19	25	19	26	2.0%	1.04 [0.75, 1.43]	2014
Tang, Y.2014	43	47	37	44	4.1%	1.09 [0.93, 1.27]	2014
**Subtotal (95% CI)**		**72**		**70**	**6.1%**	**1.07 [0.93, 1.24]**	
Total events	62		56				
Heterogeneity: Chi² = 0.07, df = 1 (P = 0.79); I² = 0%							
Test for overall effect: Z = 0.93 (P = 0.35)							
**Aidi injection plus DL VS DL**							
Xing,G.2014	52	72	31	63	3.5%	1.47 [1.10, 1.96]	2014
**Subtotal (95% CI)**		**72**		**63**	**3.5%**	**1.47 [1.10, 1.96]**	
Total events	52		31				
Heterogeneity: Not applicable							
Test for overall effect: Z = 2.60 (P = 0.009)							
**Aidi injection plus DN VS DN**							
Gao,Y.2016	36	50	27	50	2.9%	1.33 [0.98, 1.82]	2016
**Subtotal (95% CI)**		**50**		**50**	**2.9%**	**1.33 [0.98, 1.82]**	
Total events	36		27				
Heterogeneity: Not applicable							
Test for overall effect: Z = 1.83 (P = 0.07)							
**Aidi injection plus docetaxel VS Docetaxel**							
Xing, H. 2011	27	35	25	35	2.7%	1.08 [0.82, 1.42]	2011
Wang,L.2015	44	60	42	60	4.5%	1.05 [0.84, 1.31]	2015
**Subtotal (95% CI)**		**95**		**95**	**7.1%**	**1.06 [0.89, 1.26]**	
Total events	71		67				
Heterogeneity: Chi² = 0.03, df = 1 (P = 0.87); I² = 0%							
Test for overall effect: Z = 0.65 (P = 0.52)							
**Total (95% CI)**		**1337**		**1327**	**100.0%**	**1.17 [1.12, 1.22]**	
Total events	1104		936				
Heterogeneity: Chi² = 23.73, df = 32 (P = 0.85); I² = 0%							
Test for overall effect: Z = 7.59 (P < 0.00001)							
Test for subgroup differences: Chi² = 5.64, df = 5 (P = 0.34), I² = 11.3%							

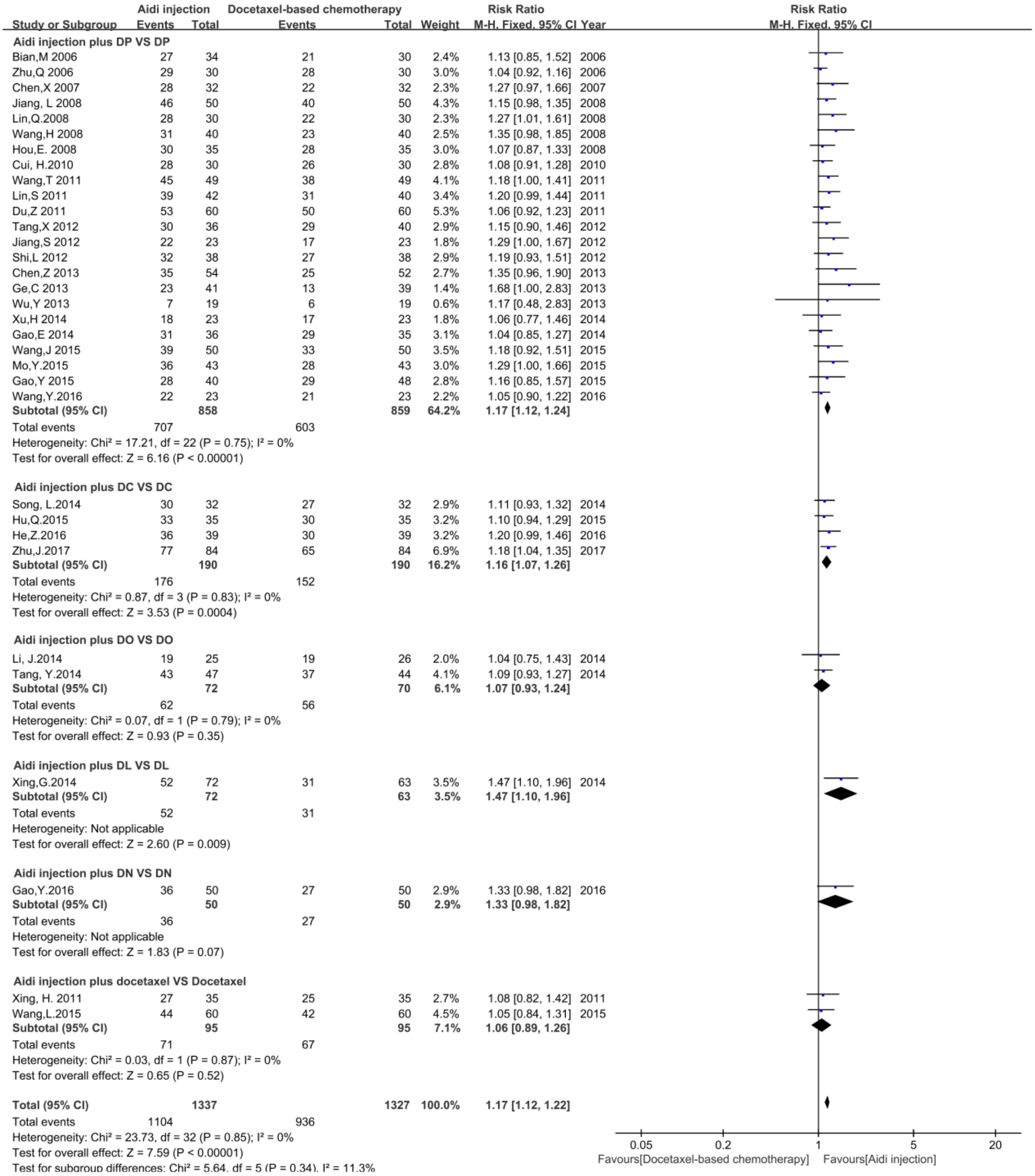

Figure S11. Subgroup analysis of DCR via docetaxel-based chemotherapy

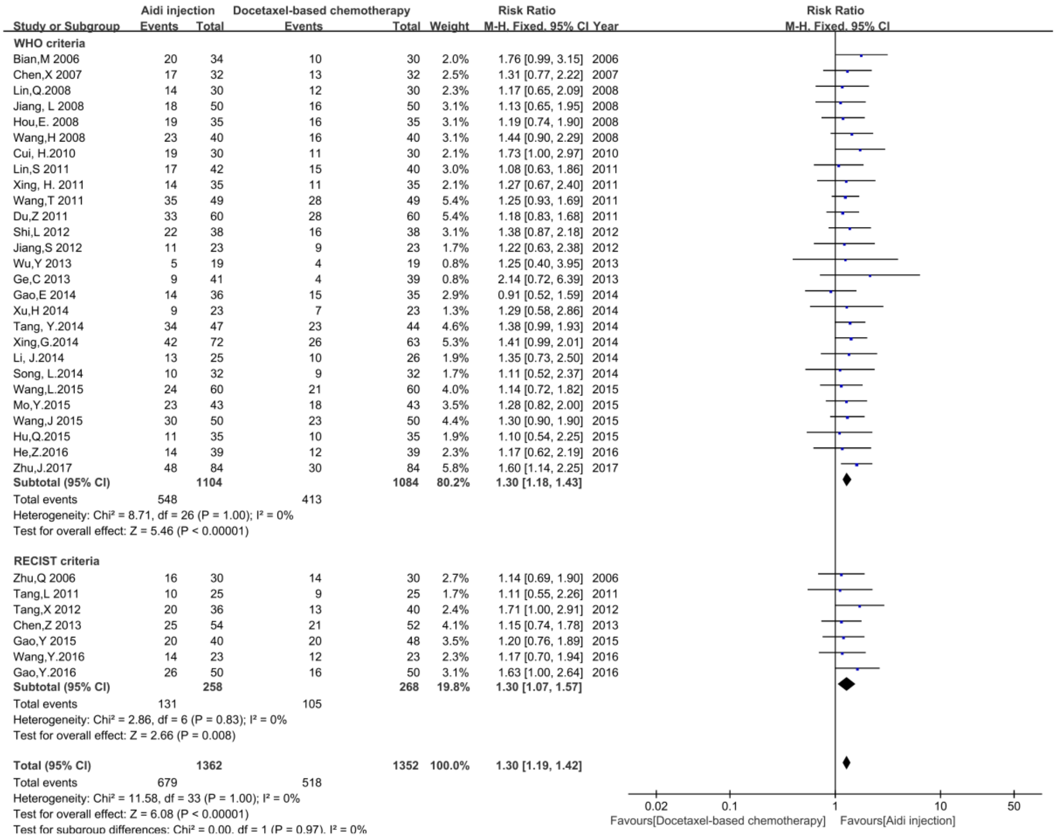

Figure S12. Subgroup analysis of ORR via evaluation criteria

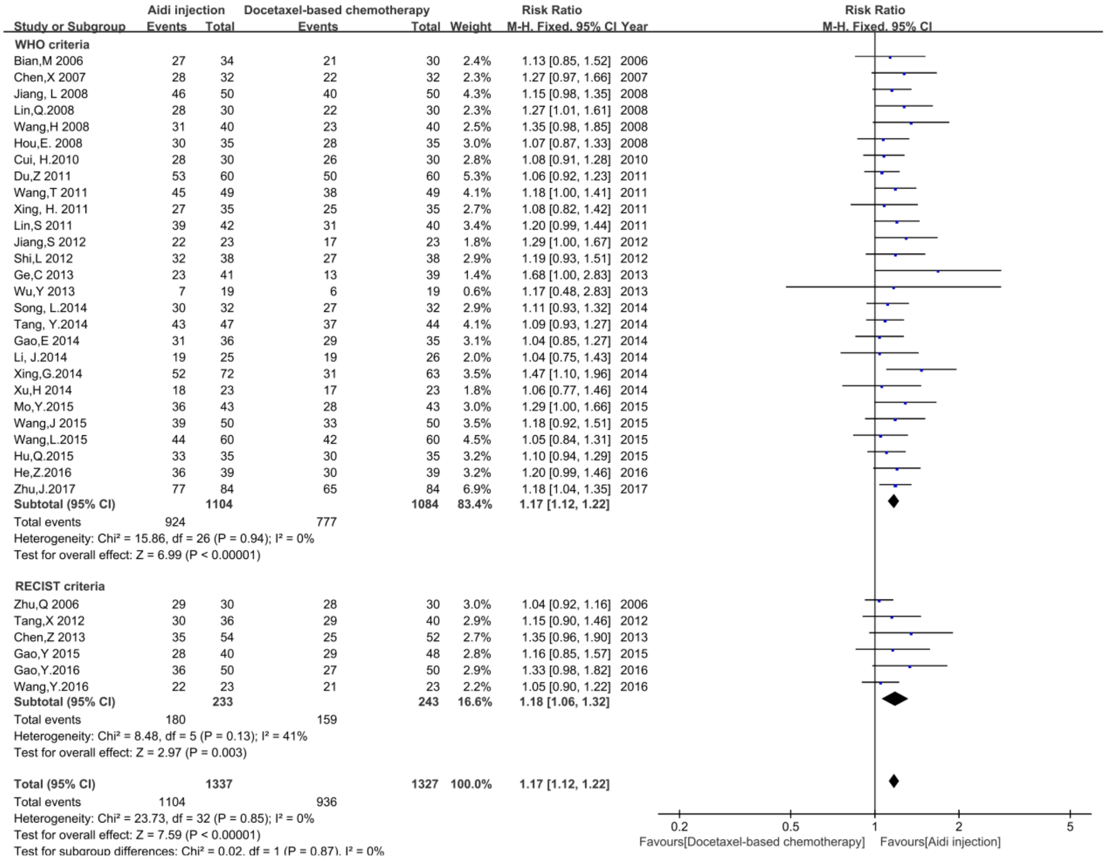

Figure S13. Subgroup analysis of DCR via evaluation criteria
